# Loss of RREB1 in pancreatic beta cells reduces cellular insulin content and affects endocrine cell gene expression

**DOI:** 10.1007/s00125-022-05856-6

**Published:** 2023-01-12

**Authors:** Katia K. Mattis, Nicole A. J. Krentz, Christoph Metzendorf, Fernando Abaitua, Aliya F. Spigelman, Han Sun, Jennifer M. Ikle, Swaraj Thaman, Antje K. Rottner, Austin Bautista, Eugenia Mazzaferro, Marta Perez-Alcantara, Jocelyn E. Manning Fox, Jason M. Torres, Agata Wesolowska-Andersen, Grace Z. Yu, Anubha Mahajan, Anders Larsson, Patrick E. MacDonald, Benjamin Davies, Marcel den Hoed, Anna L. Gloyn

**Affiliations:** 1 Oxford Centre for Diabetes, Endocrinology and Metabolism, University of Oxford, Oxford, UK; 2 Wellcome Centre for Human Genetics, University of Oxford, Oxford, UK; 3 Division of Endocrinology, Department of Pediatrics, Stanford School of Medicine, Stanford University, Stanford, CA USA; 4 Beijer Laboratory and Department of Immunology, Genetics and Pathology, Uppsala University and SciLifeLab, Uppsala, Sweden; 5 Department of Pharmacology, University of Alberta, Edmonton, AB Canada; 6 Alberta Diabetes Institute, University of Alberta, Edmonton, AB Canada; 7 Present Address: Clinical Trial Service Unit and Epidemiological Studies Unit, Nuffield Department of Population Health, University of Oxford, Oxford, UK; 8 Present Address: Genentech, South San Francisco, CA USA; 9 Department of Medical Sciences, Clinical Chemistry, Uppsala University, Uppsala, Sweden; 10 Oxford NIHR Biomedical Research Centre, Churchill Hospital, Oxford, UK

**Keywords:** Beta cell, CRISPR/Cas9, Diabetes, Differentiation, Human genetics, Pancreatic islet, RREB1, Stem cell, Transcription factor, Zebrafish

## Abstract

**Aims/hypothesis:**

Genome-wide studies have uncovered multiple independent signals at the *RREB1* locus associated with altered type 2 diabetes risk and related glycaemic traits. However, little is known about the function of the zinc finger transcription factor Ras-responsive element binding protein 1 (RREB1) in glucose homeostasis or how changes in its expression and/or function influence diabetes risk.

**Methods:**

A zebrafish model lacking *rreb1a* and *rreb1b* was used to study the effect of RREB1 loss in vivo. Using transcriptomic and cellular phenotyping of a human beta cell model (EndoC-βH1) and human induced pluripotent stem cell (hiPSC)-derived beta-like cells, we investigated how loss of RREB1 expression and activity affects pancreatic endocrine cell development and function. Ex vivo measurements of human islet function were performed in donor islets from carriers of *RREB1* type 2 diabetes risk alleles.

**Results:**

CRISPR/Cas9-mediated loss of *rreb1a* and *rreb1b* function in zebrafish supports an in vivo role for the transcription factor in beta cell mass, beta cell insulin expression and glucose levels. Loss of RREB1 also reduced insulin gene expression and cellular insulin content in EndoC-βH1 cells and impaired insulin secretion under prolonged stimulation. Transcriptomic analysis of *RREB1* knockdown and knockout EndoC-βH1 cells supports RREB1 as a novel regulator of genes involved in insulin secretion. In vitro differentiation of *RREB1*^KO/KO^ hiPSCs revealed dysregulation of pro-endocrine cell genes, including *RFX* family members, suggesting that RREB1 also regulates genes involved in endocrine cell development. Human donor islets from carriers of type 2 diabetes risk alleles in *RREB1* have altered glucose-stimulated insulin secretion ex vivo, consistent with a role for RREB1 in regulating islet cell function.

**Conclusions/interpretation:**

Together, our results indicate that RREB1 regulates beta cell function by transcriptionally regulating the expression of genes involved in beta cell development and function.

**Graphical abstract:**

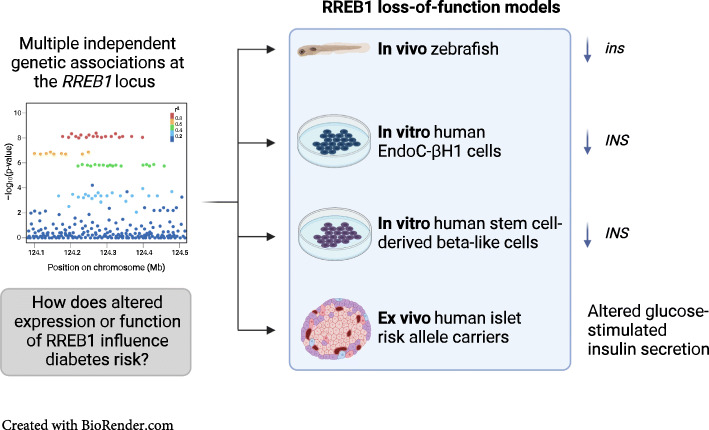

**Supplementary Information:**

The online version contains peer-reviewed but unedited supplementary material available at 10.1007/s00125-022-05856-6.

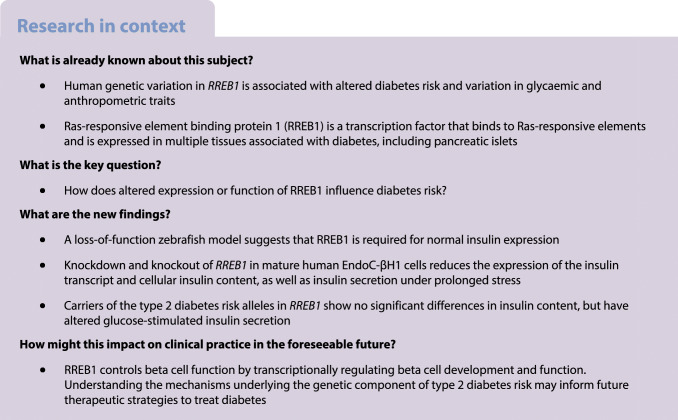



## Introduction

Genome-wide association studies have discovered multiple independent signals at the *RREB1/SSR1* locus that are associated with altered type 2 diabetes risk and various metabolic and anthropometric traits, including fasting glucose levels and height [1–4]. Genetic fine-mapping identified the coding variant rs9379084 (p.Asp1171Asn) as causal (92% posterior probability for type 2 diabetes), strongly supporting a role for *RREB1* as the effector transcript at this locus [5]. Carriers of the minor allele encoding p.Asn1171-*RREB1*, predicted to have a detrimental effect on Ras-responsive element binding protein 1 (RREB1) function (CADD score 28.2), have a lower risk of developing type 2 diabetes and lower fasting glucose levels, on average [2]. The shared association between type 2 diabetes risk and quantitative measures of islet function supports the islet as a key tissue mediating disease [6–8] and suggests a potential role for RREB1 in beta cell development and/or function.

Although RREB1 has been studied in several different cellular contexts, there have been no investigations into its role in the pancreatic beta cell [9–11]. *RREB1* encodes a zinc finger transcription factor that is expressed in several type 2 diabetes-relevant tissues, including pancreatic islets, adipose tissue, liver and skeletal muscle [12–14]. Several lines of evidence support a potential developmental role for RREB1: (1) homozygous deletion of *Rreb1* in mice is embryonic lethal [10]; (2) the *RREB1* transcript [15] and protein [16] are detected during in vitro endocrine cell differentiation of human induced pluripotent stem cells (hiPSCs); and (3) RREB1 is a downstream target of MAPK/ERK, a signalling pathway that is important for early human beta cell differentiation [17]. However, whether genetic variation in *RREB1* influences diabetes risk as a result of altered endocrine cell development and/or function is unknown. In this study, we explore the role of RREB1 in an in vivo zebrafish model, in authentic cellular models of human beta cells and by ex vivo characterisation of human donor islets from carriers of *RREB1* type 2 diabetes risk alleles.

## Methods

### Zebrafish studies

#### Husbandry and transgenic lines

Adult zebrafish (*Danio rerio*) were housed in systems with recirculating filtered water at 28.5°C (Aquaneering, USA) on a 14/10 h light/dark cycle. Through crossing we generated fish with transgenically expressed, fluorescently labelled pancreatic beta cell nuclei (*Tg(ins:Hsa.HIST1H2BJ-mCherry)*^*vu513*^ (Vanderbilt University, USA [18]) and hepatocytes (*Tg(-2.8fabp10a:EGFP)*^*as3TG*^) (European Zebrafish Resource Center [EZRC] [19, 20]) in the AB background (EZRC; http://zfin.org/ZDB-GENO-960809-7). Further details on validation of the *Tg(ins:Hsa.HIST1H2BJ-mCherry)*^*vu513*^ reporter can be found in electronic supplementary materials (ESM) Methods. All zebrafish handling and experiments were carried out in agreement with Swedish animal welfare laws and were approved by the Ethical Committee for Animal Research of the Swedish Ministry of Agriculture (Dnrs C14/16 and 5.8.18-13680/2020).

#### Sequence analysis

The human RREB1 amino acid sequence and the amino acid sequences of the zebrafish orthologues (rreb1a and rreb1b; ESM Table 1) were downloaded from uniprot (www.uniprot.org). Using MEGA11 software (https://www.megasoftware.net, version 11.0.10 [21]), amino acid sequences across species were aligned by MUSCLE multiple sequence alignment and a phylogenic tree was constructed by neighbour-joining with bootstrapping (2000 replicates).

#### CRISPR/Cas9-mediated mutagenesis in zebrafish embryos

*Danio rerio* genome version GRCz11 (www.ensembl.org/index.html) was used for planning all CRISPR/Cas9-related work. The online design tool CRISPOR (v4.98 and v4.99; http://crispor.tefor.net [22]) was used to identify suitable single guide RNAs (sgRNAs) in the coding regions of the *rreb1a* (ENSDARG00000063701) and *rreb1b* (ENSDARG00000042652) genes that (1) target early exons (first quarter of the coding regions); (2) are shared across all relevant transcripts of *rreb1a* and *rreb1b*; (3) have a high ‘azimuth score’; and (4) have no or very few predicted off-targets, with zero to four mismatches (ESM Table 2). As a control gene, we targeted *kita* (ENSDARG00000043317) using a sgRNA designed following the above criteria, which was identical to CRISPR1-kita (ZDB-CRISPR-180314-3; www.zfin.org) (ESM Table 2). The genes *rreb1a*, *rreb1b* and *kita* (*rreb1a/b* crispants) or *kita* only (control) were targeted using the Alt-R CRISPR/Cas9 system (IDT, Belgium) [23]. crRNAs with the selected target sequences and Alt-R CRISPR/Cas9 tracrRNA (cat no. 072533, IDT) were resuspended at 100 μmol/l in Duplex Buffer and stored at –20°C until use. A total of 50 μmol/l sgRNA was prepared by mixing equal volumes of 100 μmol/l crRNA and 100 μmol/l tracrRNA, followed by annealing for 5 min at 95°C, cooling at 0.1°C/s to 25°C, incubating for 5 min at 25°C and rapid cooling to 4°C (stored at –20°C until use). Injection mixes were prepared fresh on the day of microinjections by mixing 1 μl *kita* sgRNA, 1 μl *rreb1a* sgRNA and 1 μl *rreb1b* sgRNA with 2.4 μl Alt-R Cas9 (IDT) and 4.6 μl ultra-pure H_2_O for crispants; or 1 μl *kita* sgRNA, 0.8 μl Alt-R Cas9 and 7.2 μl ultra-pure H_2_O for control experiments. Mixes were incubated at 37°C for 5 min and 1 μl of phenol red (Sigma-Aldrich, Merck, Sweden) was added as a visual injection aid. For microinjections, eggs from all clutches of the same round of crossings of several adults were mixed and then randomised into two groups to generate *rreb1a*/*b* crispants and controls. Microinjections into the cell or into the yolk close to the cell were performed at the single-cell stage using standard microinjection equipment.

At 1 day post fertilisation (dpf), dead embryos and unfertilised eggs were removed and the remaining embryos were aliquoted at 50–60 embryos per Petri dish. Eggs and embryos were kept in filtered water with methylene blue until 5 dpf. At 4 or 5 dpf, the success rate of CRISPR/Cas9 gene editing was assessed by optically checking for lack of pigmentation using a stereo microscope, as loss of *kita* results in absence and/or reduced migration of melanocytes [24, 25]. Larvae with pigmentation were discarded. Across the individual experiments performed to reach the final sample size, 116–424 larvae per experiment survived to day 5. The mean±SD survival rate from 1 to 5 dpf was 41±18% in *rreb1a/b* crispants and 64±13% in controls. While this reflects a significant difference in survival rate between *rreb1a/b* crispants and controls (*p*=2×10^–3^), the difference is not dissimilar to what we observed across ongoing experiments for 67 candidate genes for cardiometabolic diseases targeted the same way, with mean survival rates of 37% in crispants for candidate genes and 58% in controls. Thus, mutations in *rreb1a* and *rreb1b* are not more harmful for early-stage development in zebrafish than mutations in other candidate genes associated with cardiometabolic diseases.

At 5 dpf, non-melanised embryos from each group were placed in experimental tanks at a ratio of *rreb1a/b* crispants to controls of 70:30, at 30 or 60 embryos per tank in 300 or 600 ml of filtered water, respectively, to reduce the risk of tank-specific effects. In addition, mixing controls and *rreb1a/b* crispants served as a blinding method during all experimental procedures that followed (feeding and distributing larvae across plates, imaging, sample preparation and biochemical analysis). Larvae were fed twice daily at approximately 09:00 and 15:30 using a standardised amount (16 mg/30 larvae) of regular dry food per feeding (zebrafeed <100 μm; SPAROS, Portugal). Feeding began on the afternoon of day 5 and was continued until the afternoon of day 9. Full water exchanges were performed midday at 7 and 9 dpf. The experiment was performed a total of six times to reach the final sample size.

While we were able to target all four putative transcripts of the zebrafish *rreb1a* with a single sgRNA, this was not possible for *rreb1b*. For *rreb1b*, we were able to target two major transcripts that code for proteins of 1499 and 1671 amino acids, while the putative transcript *rreb1b-202* could not be targeted. However, this transcript has only the 5′ untranslated region in common with one of the two major transcripts and its coding region encodes a peptide that is only 29 amino acids long and does not align with any of the other amino acid sequences. Therefore, we conclude that *rreb1b-202* does not code for a functional *rreb1b* isoform and does not need to be targeted. It may still be involved in regulatory functions, but these were not the focus of this study.

#### Imaging of zebrafish larvae

Imaging of zebrafish larvae was performed at 10 dpf. On the morning of day 10, live zebrafish larvae were washed twice with filtered water, followed by incubation for 30 min at 28.5°C in 12.5 μmol/l monodansylpentane (cat. no. SM1000a, Abcepta, USA) [26] in PBS (0.8 ml PBS per larva), to label neutral lipids. Next, larvae were anaesthetised by adding tricaine to a final concentration of 230 μmol/l and were then placed in 96-well plates. From here they were automatically aspirated and oriented in a glass capillary using an Autosampler and Vertebrate Automated Screening Technology (VAST) BioImager (Union Biometrica, Belgium) built on the stage of a Leica DM6000B fluorescence microscope with a Leica DFC 365 FX CCD camera (Micromedic, Sweden). For each larva, 12 full body images were first acquired, one every 30° of rotation around the longitudinal axis of the body using the VAST BioImager’s bright-field camera. Optical sections of the pancreatic islet (TexasRed filter set, HCX APO LU-V-I 40×/0.80 water immersion objective, 45 images/stack, 66.04 μm Z-size) and liver (GFP and CFP filter sets, HC APO LU-V-I 10×/0.3 water immersion objective, 35 images/stack, 51.03 μm Z-size) were then acquired using the fluorescence microscope. After imaging, larvae were dispensed back into 96-well plates, euthanised by prolonged exposure to tricaine and kept on ice. Water was removed and samples were stored at –20°C until further analysis. Relevant traits for body size (whole-body length, dorsal area, lateral area), pancreatic diabetes-related traits (number of insulin-expressing nuclei as a proxy for beta cell mass, mean and total nuclear volume of insulin-expressing cells, mean fluorescence intensity of insulin-expressing nuclei as a proxy for beta cell insulin expression [ESM Fig. 1], islet dimensions) and hepatic diabetes-related traits (liver area, number and size of lipid objects) were quantified in imaging data using custom-written deep-learning algorithms.

#### Glucose and lipid quantification

Imaged larvae or larvae raised to 10 dpf under the same conditions but collected at 09:00 without having been imaged because of time constraints were stored at –20°C until further processing. Single larvae per well of a 96-well plate were homogenised with a 1.4 mm acid-washed zirconium bead (OPS Diagnostics, USA) in 88 μl ice-cold PBS using a MiniG 1600 homogeniser (SPEX SamplePrep, USA). Samples were centrifuged for 5 min at 3500 *g* at 4°C. The supernatant was stored at –80°C until further processing, while the remaining pellet was kept to isolate DNA. Concentrations of glucose, LDL-cholesterol, triacylglycerol and total cholesterol were quantified using enzymatic assays and a Mindray Analyzer (Mindray, China), as described previously [27].

#### Identification of *rreb1a/b* crispant and control zebrafish larvae by fragment length analysis and quantitative real-time PCR

The *rreb1a/b* crispant and control larvae were categorised by fragment length analysis (as described by Varshney et al [28]), using DNA extracted from the pellets remaining after homogenisation and centrifugation of larvae. Briefly, the remaining pellets were digested using 200 μg/ml proteinase K (ThermoFisher Scientific, Sweden) in 50 μl lysis buffer per larva (10 mmol/l TRIS-HCl pH 8, 50 mmol/l KCl, 0.3% vol/vol Tween 20, 0.3% vol/vol Igepal, 1 mmol/l EDTA; Sigma-Aldrich, Sweden). Samples were incubated at 55°C for 2 h and at 95°C for 10 min after which insoluble particles were removed by centrifugation. The *rreb1a* and *rreb1b* amplicons covering the target regions of the sgRNAs were amplified by quantitative real-time PCR (qPCR) in separate reactions using primers (ESM Table 3) at a final concentration of 200 nmol/l with either platinum Taq (ThermoFisher Scientific, Sweden) or OneTaq (New England Biolabs, BioNordika Sweden, Sweden), following the manufacturers’ protocols. To ascertain how well fragment length analysis quantified CRISPR/Cas9-induced mutagenesis, qPCR was additionally performed in a subset of samples (*n*=158) using the primers described in ESM Table 4.

PCR products were diluted from 1:10 to 1:20 before mixing 1.5 μl of diluted sample with 10 μl Hi-Di buffer (containing 73.3×diluted GS400HD ROX standard; ThermoFisher Scientific, Sweden), followed by denaturation at 95°C for 5 min, rapid cooling on ice and capillary electrophoresis on a DNAnalyzer (3730xl, ThermoFisher Scientific, Sweden). Chromatograms were analysed using Peak Scanner software (v1.0 and v2.0; ThermoFisher Scientific, Sweden) and fragment lengths were exported for further analysis with a custom RStudio (v1.4.1103-4 to v1.4.1717-3) [29] markdown script in R (v4.0.4 to v4.1.0) [30] that calculates the relative area of the peak at the wild-type allele’s fragment length relative to areas of any other peaks within ±50 base pairs of the wild-type peak. PCR products generated using DNA of uninjected larvae from the same crossing were used to experimentally determine the fragment length and relative peak area of the *rreb1a* and *rreb1b* wild-type PCR products.

To ascertain how well fragment length analysis managed to quantify CRISPR/Cas9-induced mutagenesis, we additionally used DNA from a subset of experimental larvae (*n=*126) and uninjected control larvae (*n=*32) in a qPCR-based analysis [31]. qPCR was performed in duplicate per sample, in 10 μl reactions using 1–2 μl of 10–20× diluted DNA as a template and 200–400 nmol/l primers (ESM Table 3) with the PowerUP SYBR Green Master Mix (ThermoFisher Scientific, Sweden) in an AriaMx Real-Time PCR System (Agilent Technologies, USA). Dilution series of samples from uninjected siblings were used as a reference for relative quantification. Two non-targeted loci in *rreb1a* and *rreb1b* were used for relative quantification of genomic DNA and normalisation of quantification data from the on-target primer pairs. Based on the congruence across qPCR and fragment length analysis results, samples were assigned to the control group if >70% of the *rreb1a* peak area and >60% of the *rreb1b* peak area were wild-type and to the *rreb1a/b* crispant group if ≤60% and ≤50% of peak areas were wild-type for *rreb1a* and *rreb1b*, respectively (ESM Fig. 2). Larvae that did not fulfil either criterion were excluded from the analysis. Across all six rounds of the experiment, a total of 175 larvae were imaged at 10 dpf and 49 additional larvae at 10 dpf were phenotypically characterised using biochemistry only, because of time constraints on the day of imaging. Of these 224 larvae, 92 were *rreb1a/b* crispants, 64 were controls and 68 had inconclusive fragment length analysis results and were excluded from the analysis.

### EndoC-βH1 cells

#### Routine cell culture

EndoC-βH1 cells (Human Cell Design, France) [32] were grown in DMEM (low glucose, pyruvate) supplemented with 2% wt/vol Bovine Serum Albumin Fraction V, fatty acid free (Roche, USA/UK), 50 μmol/l 2-mercaptoethanol, 10 nmol/l nicotinamide (Sigma-Aldrich, USA/UK), 5.5 μg/ml transferrin (Sigma-Aldrich, USA/UK), 6.6 ng/ml sodium selenite (Sigma-Aldrich, USA/UK), 100 U/ml penicillin and 100 µg/ml streptomycin and 2 mmol/l l-glutamine on cell culture plates coated with DMEM (high glucose) supplemented with 1% vol/vol extracellular matrix (Sigma-Aldrich, USA/UK), 2 μg/ml fibronectin (Sigma-Aldrich, USA/UK) and 100 U/ml penicillin and 100 µg/ml streptomycin at 37°C and 5% CO_2_. Cell culture reagents were manufactured by ThermoFisher Scientific (USA/UK) unless otherwise stated. All EndoC-βH1 cell lines were routinely tested and were negative for mycoplasma.

#### EndoC-βH1 gene silencing

Gene silencing was performed according to the Lipofectamine RNAiMAX transfection protocol using 25 nmol/l SMART pool (mixture of four siRNAs) ON-TARGETplus siRNAs (siNT [non-targeting negative control]: D-001810-10-05, si*RREB1*: L-019150-00-0005, si*RFX2*: L-011129-00-0005, and si*RFX3*: L-011764-00-0005; PerkinElmer, USA/UK) diluted in Opti-MEM reduced serum-free medium and 0.4% vol/vol RNAiMAX (ThermoFisher Scientific, USA/UK). Silencing efficiency was assessed 96 h after transfection by qPCR and/or western blot. For dual gene silencing experiments, 25 nmol/l of si*RFX2* and si*RFX3* were compared with 25 nmol/l of siNT, si*RFX2* or si*RFX3* alone. qPCR was performed 4 days post transfection.

#### CRISPR/Cas9-mediated generation of *RREB1*-KO EndoC-βH1 cells

A non-clonal, pooled *RREB1*-KO EndoC-βH1 cell line was generated using sgRNAs from the Toronto KnockOut (TKO) Library (v3) [33] or sgRNAs designed with the CRISPOR online design tool (v4.0; http://crispor.tefor.net) [34]. sgRNA oligonucleotides targeting *RREB1* exon 4 (ATGACGTCAAGTTCGCCCGC), exon 5 (AGTGCAAATCTTCTCACACA), exon 8 (GTATGGACTGGAGACCCACA) and exon 12 (GACAGACTCCCCCAAAAGCG) were amplified and subcloned into the BsmBI restriction enzyme sites in the lentiviral vector pLentiCRISPRv2 [35]. Lentiviruses were produced by Lenti-X HEK293T cells (Takara Bio, Japan) co-transfected with 6.85 μg of pMD2.G (RRID:Addgene_12259), 10.3 μg of psPAX2 (RRID:Addgene_12260) and 12.85 μg pLentiCRISPRv2-sgRNAs. To calculate functional viral titre, serial dilutions (1:50 to 1:6400) of virus were added to EndoC-βH1 cells. Following 7 days of puromycin selection, cell viability was determined using the CyQUANT Direct Cell Proliferation Assay (cat. no. C35012, ThermoFisher Scientific, UK) and cell count was compared with that for unselected control cells. The functional viral titre was calculated by the volume of virus needed to infect 26% of cells, which is a multiplicity of infection (MOI) of 0.3 under a simplified Poisson distribution [36]. EndoC-βH1 cells were transduced at a MOI of 10 and selected with 4–6 μg/μl puromycin for 7 days. Estimations of sgRNA cutting efficiency and the frequency of insertions and deletions generated were performed using TIDE analysis (Tracking of Indels by DEcomposition; http://shinyapps.datacurators.nl/tide/) [37]. A control EndoC-βH1 cell line was generated in parallel using a Cas9-only (without sgRNA) lentivirus (*RREB1*-EV).

#### EndoC-βH1 insulin secretion assays

Static insulin secretion assays were performed at basal (1 or 2.8 mmol/l) and high (16.7 or 20 mmol/l) glucose as previously described [38]. Cellular insulin content was extracted using ice-cold acid ethanol (1.5% vol/vol concentrated HCl, 75% vol/vol ethanol and 23.5% vol/vol deionised water). The Insulin (human) AlphaLISA Detection Kit (PerkinElmer) was used to measure the amount of secreted insulin (supernatants) and cellular insulin content. Samples were diluted in 1×AlphaLISA immunoassay buffer (supernatant 1:10, cellular insulin content 1:50) and analysis was performed in 96-well white 1/2 area plates on an EnSpire plate reader (PerkinElmer). Sample values were interpolated from an insulin analyte standard curve included on every plate using a four-parameter non-linear regression of log-transformed insulin count data in Prism 8 (GraphPad Software, USA). For forskolin-mediated insulin depletion assays, cells were incubated in cell culture media supplemented with 20 mmol/l glucose and 10 μmol/l forskolin for 30 min and allowed to recover in 2.8 mmol/l glucose for a further 30 min before measuring glucose-stimulated insulin secretion. Insulin content was measured in picograms and normalised to the number of cells plated (pg/cell). Insulin secretion was expressed as raw insulin released into culture media (pg/ml).

### Human induced pluripotent stem cells

#### Routine cell culture

The hiPSC cell line SB Ad3.1 was obtained from the StemBANCC consortium via the Human Biomaterials Resource Centre, University of Birmingham [39]. Cells were grown in mTeSR1 basal medium supplemented with mTeSR1 5× supplement (Stemcell Technologies, UK), 100 U/ml penicillin and 100 μg/ml streptomycin on cell culture plates coated with DMEM/F12 (Sigma-Aldrich, UK) supplemented with hESC-qualified Matrigel, diluted according to the manufacturer’s instructions and lot number (Corning, UK). All cell lines were maintained at 37°C and 5% CO_2_ and routinely tested negative for mycoplasma.

#### Genome editing of hiPSCs

The sgRNAs directed to *RREB1* exon 4 (GTCAAGTTCGCCCGCTGGCT) and exon 10 (ACCCCGCGCCAACAGCGGCG) were designed using the Massachusetts Institute of Technology (MIT) CRISPR online design tool (previously available at http://crispr.mit.edu) and cloned into the BsbI restriction enzyme site of the plasmid pX330-U6-Chimeric_BB-CBh-hSpCas9 [40] as previously described [41]. As the SB hiPSC cell line is heterozygous for the type 2 diabetes-protective (p.Asn1171) allele, genome editing was used to generate *RREB1*^WT/WT^ clones with a 141 nucleotide single-stranded oligodeoxynucleotide repair template (Eurogentec, Belgium) containing (1) the type 2 diabetes risk allele (c.3511G, p.Asp1171); (2) a silent mutation to introduce a HincII restriction enzyme site at codon 1170 (c.3510G>C) for genotyping; and (3) a silent mutation in the protospacer adjacent motif (PAM) sequence (c.3507G>C) located in exon 10. Cells were co-transfected either with 1 μg of pX330-Puro-Cas9 plasmid containing exon 10 sgRNA and 100 nmol/l of the repair template (to generate *RREB1*^WT/WT^) or with 1 μg pX330-Puro-RREB1 plasmids containing exon 4 and exon 10 sgRNAs (to generate *RREB1*^KO/KO^) using FuGENE 6 (Promega, UK). Transfected cells were selected with 400 ng/ml of puromycin-containing mTeSR1 media for 48 h. Selection media was then removed and cells were grown in antibiotic-free mTeSR1 until ∼90% confluency. Cells were then replated at low density (2000 cells/60 mm dish), allowed to form clones and picked using a microscope-mediated pipetting approach into individual wells of a 96-well plate containing mTeSR1 and 10 μmol/l Y-27632 (Stemcell Technologies). After approximately 7 days, colonies were split into two replica Matrigel-coated 96-well plates for either expansion or genotyping. The resulting clonal cell lines did not have any of the ten most common coding mutations in the *TP53* gene (ESM Table 5) and had a normal karyotype, both of which can be affected by the genome editing pipeline.

#### In vitro hiPSC differentiation towards beta-like cells

For differentiation of genome-edited hiPSC cell lines towards beta-like cells (BLCs), hiPSCs were plated in 12-well CellBind plates coated with growth factor-reduced Matrigel (1:30; Corning, UK) at an optimised density of 0.8–1.3×10^6^ cells/well in mTeSR1 supplemented with 10 μmol/l Y-27632 (Stemcell Technologies). Once cells had attached (6+ h), media containing Y-27632 was removed and replaced with mTeSR1. In vitro differentiation was started 24 h after plating following the protocol of Rezania et al [42]. Basal and complete differentiation media are described in ESM Table 6.

#### Flow cytometry

Genome-edited hiPSCs were evaluated for expression of pluripotency markers using the Human Pluripotent Stem Cell Transcription Factor Analysis Kit (BD Biosciences, UK). Quantification of 5-ethynyl-2′-deoxyuridine (EdU)+hiPSCs was performed following 30 min of EdU exposure using the Click-iT EdU Alexa Fluor Assay Kit (ThermoFisher Scientific, UK). In vitro differentiation efficiency was assessed by expression of stage-specific markers of definitive endoderm (CXCR4: 1:40, cat. no. FAB173P, RRID: AB_357083, R&D Systems, USA) and BLCs (PE Mouse Anti-NKX6.1: 1:40, cat. no. 563023, RRID:AB_2716792; AF647 Mouse Anti-C-Peptide: 1:200, cat. no. 565831, RRID:AB_2739371; both BD Biosciences). Cells were dissociated into a single-cell suspension, fixed with Cytofix Fixation Buffer (BD Biosciences) or 4% wt/vol paraformaldehyde (ThermoFisher Scientific, UK), permeabilised in Perm/Wash Buffer or Phosflow Perm Buffer III (BD Biosciences) and stained for cell surface or intracellular markers. Dead cells were excluded using the LIVE/DEAD Fixable Violet Dead Cell Stain Kit for 405 nm excitation (ThermoFisher Scientific, UK). Samples were analysed on either the SH800 Cell Sorter (Sony, USA) or the FACSCanto II (BD Biosciences). Data analysis was performed using FlowJo 10.6.0 (https://www.flowjo.com).

### Gene expression

#### RNA extraction and sequencing

For RNA extraction, cells were lysed in TRIzol reagent (ThermoFisher Scientific, UK) and processed according to the Direct-zol RNA Miniprep Kit manual (Zymo Research, UK). The NEBNext PolyA mRNA Magnetic Isolation Module (New England Biolabs, UK) was used for isolation of polyadenylated transcripts. RNA-seq libraries were prepared using the NEBNext Ultra Directional RNA Library Prep Kit with 12 cycles of PCR and custom 8 bp indexes (New England Biolabs). Libraries were multiplexed and sequenced on an Illumina HiSeq4000 as 75-nucleotide paired-end reads.

#### Differential expression and functional enrichment analysis

Reads were mapped to the human genome build hg19 using STAR (v2.5) [43]. GENCODE (v19; https://www.gencodegenes.org/human/release_19.html) was used as a transcriptomic reference [44]. Quantification of gene expression was performed by featureCounts from the Subread package (v1.5; http://subread.sourceforge.net/) [45]. To adjust for technical effects, removal of unwanted variation was conducted in R (v3.3.3) using the RUVSeq following the instructions in the manual compiled on 2 May 2019 [46]. Read counts were filtered to include only genes that reached one transcript per million in at least one cell line and in at least one stage before normalisation. Differential gene expression analysis was conducted in R using the Bioconductor package DESeq2 [47] according to the vignette compiled on 30 November 2016 and significance threshold of *q*<0.01. Differentially expressed genes (DEGs) were evaluated for enrichment in gene ontology terms (gene ontology biological processes, Kyoto Encyclopedia of Genes and Genomes [KEGG] and Reactome pathways) using g:Profiler with the significance threshold set to *q*<0.01 using the tailor-made g:SCS algorithm for multiple testing (https://biit.cs.ut.ee/gprofiler/gost) [48]. Correlation of gene expression patterns during beta cell development was calculated using the weighted gene co-expression network analysis (WGCNA) R software package (v1.51) [49, 50]. Transcription factor binding motif enrichment analysis was performed using the iRegulon (v1.3) Cytoscape (v3.7.0) plugin [51]. To identify key transcription factors mediating gene expression variation, motif activity response analysis (MARA) was performed using the online tool ISMARA (Integrated System for MARA) [52].

#### Quantitative real-time PCR

TaqMan PCR assays were used to measure *RREB1* (Hs00366111_m1), *INS* (Hs00355773_m1), *CAMK2A* (Hs00392405_m1), *GPR56* (Hs00173754_m1), *RFX2* (Hs00172177_m1), *RFX3*, (Hs01060440_m1) and *TBP* (Hs00427620_m1) gene expression (ThermoFisher Scientific, UK). qPCR reactions were performed on a 7900HT Fast Real-Time PCR System (ThermoFisher Scientific, UK) using SDS software (v2.3; Applied Biosystems) and the following conditions: 50°C for 2 min and 95°C for 10 min followed by 40 cycles of 95°C for 15 s and 60°C for 1 min. Cycle thresholds were transformed to gene copy numbers and normalised to the geometric mean of the housekeeping genes *TBP* and *PPIA*, except for Fig. 7, in which gene expression was normalised to *TBP* only.

### Protein detection

#### Immunoblotting

Cells were collected using Trypsin/EDTA solution or TrypLE Select and lysed in pre-chilled whole cell extraction buffer (20 mmol/l HEPES pH 7.8, 0.42 mol/l NaCl, 0.5% vol/vol NP-40, 25% glycerol vol/vol, 0.2 mmol/l EDTA pH 8, 1.5 mmol/l MgCl_2_) supplemented with 1 mmol/l DTT (ThermoFisher Scientific, UK) and 1× protease inhibitor cocktail (Sigma-Aldrich, UK). Protein lysates were quantified using Bradford Assay Reagent, run on a 4–20% Criterion TGX Stain-Free Precast Gel and transferred to 0.2 μmol/l PVDF membrane (Bio-Rad Laboratories, UK). Primary antibodies against FLAG (1:10,000, cat. no. F3165, RRID:AB_259529), RREB1 (1:500, cat. no. HPA001756, RRID:AB_1856477), RFX2 (1:1000, cat. no. HPA048969, RRID:AB_2680577), RFX3 (1:1000, cat. no. HPA035689, RRID:AB_10671224), RFX6 (1:1000, cat. no. HPA037696) (all Sigma-Aldrich, UK), Cas9 (1:1000, cat. no. sc-517386, RRID:AB_2800509) or β-tubulin (1:2000, cat. no. sc-365791, RRID:AB_10841919) (both Santa Cruz Biotechnology, USA) were used, followed by horseradish peroxidase (HRP)-conjugated IgG secondary antibodies (1:2500, ThermoFisher Scientific, UK). Chemiluminescent signals were detected using Clarity Western Enhanced Chemiluminescence Substrate (Bio-Rad Laboratories, UK) and visualised on a ChemiDoc MP. All antibodies were validated in samples lacking protein expression or siRNA-treated samples.

#### Immunofluorescence staining

Cells were fixed with 4% vol/vol paraformaldehyde, permeabilised in 0.001% vol/vol Triton X-100 (Sigma-Aldrich, UK) and blocked in 5% vol/vol swine serum. Cells were incubated with primary antibodies to RREB1 or β-tubulin (1:100) at 4°C overnight. The following day, cells were washed before incubation with Alexa Fluor-conjugated secondary antibodies in 5% vol/vol swine serum in PBS (1:100, cat. no. A-21206, RRID:AB_2535792 and cat. no. A-21435, RRID:AB_2535856; ThermoFisher Scientific, UK) and mounted in Vectashield mounting medium (Vector Laboratories, UK). Immunostained cells were visualised on a Bio-Rad Radiance 2100 confocal microscope with a 60× 1.0 numerical aperture water immersion objective. Images were acquired using LaserSharp 2000 software (Bio-Rad, UK) for three channels (green, red and far-red). For each channel, laser settings were optimised first and the same settings were used for all samples. Image files were exported using the LSM Image Browser 4.2 (Carl Zeiss, Germany).

### Human islet studies and genotyping

Donor organs from individuals without type 2 diabetes were obtained with written consent and approval of the Human Research Ethics Board of the University of Alberta (Pro00013094; Pro 00001754). Genotyping was performed on an Illumina Omni2.5Exome-8 version 1.3 BeadChip array on DNA extracted from exocrine tissue, spleen or islets if no other tissue was available. Isolation of human islets and static glucose-stimulated insulin secretion assays were performed as described in the protocols.io repository [53].

### Statistical analysis

Zebrafish data management for fragment length analysis was performed in R (v4.0.4 to v4.1.0) [30]. All downstream zebrafish data management and statistical analyses were performed using Stata MP (v16; StataCorp, USA). For zebrafish studies, although all outcomes were normally distributed, inverse normal transformations were applied before the statistical analysis to enable a comparison of effect sizes across outcomes. Genetic effects on outcomes of interest were subsequently examined using linear regression analysis, adjusting for the time of day at which larvae were imaged, the tank that larvae were in from day 5 to day 10, and the round of the experiment (from one to six) in which they were examined. Effects on dorsal and lateral body surface area were additionally adjusted for body length as a covariable; effects on whole-body glucose, LDL-cholesterol, triacylglycerol and total cholesterol levels were additionally adjusted for the position of the sample in the Mindray Analyzer, the run in which the sample was analysed and whether or not the larva had been imaged.

For human data, statistical analysis was performed in Prism 8.1.2 (GraphPad Software). Results from multiple experiments are expressed as mean±SEM. A two-tailed unpaired *t* test was used to determine *p* values for two unmatched groups following a Gaussian distribution. Multiple groups were compared using two-way ANOVA followed by Sidak’s or Tukey’s multiple comparisons test. Significance was determined at *p*<0.05. The number of biologically independent experiments (*n*) carried out are as indicated in the figure legends.

## Results

### *rreb1* loss of function in zebrafish reduces beta cell *ins* expression

**Fig. 1:**
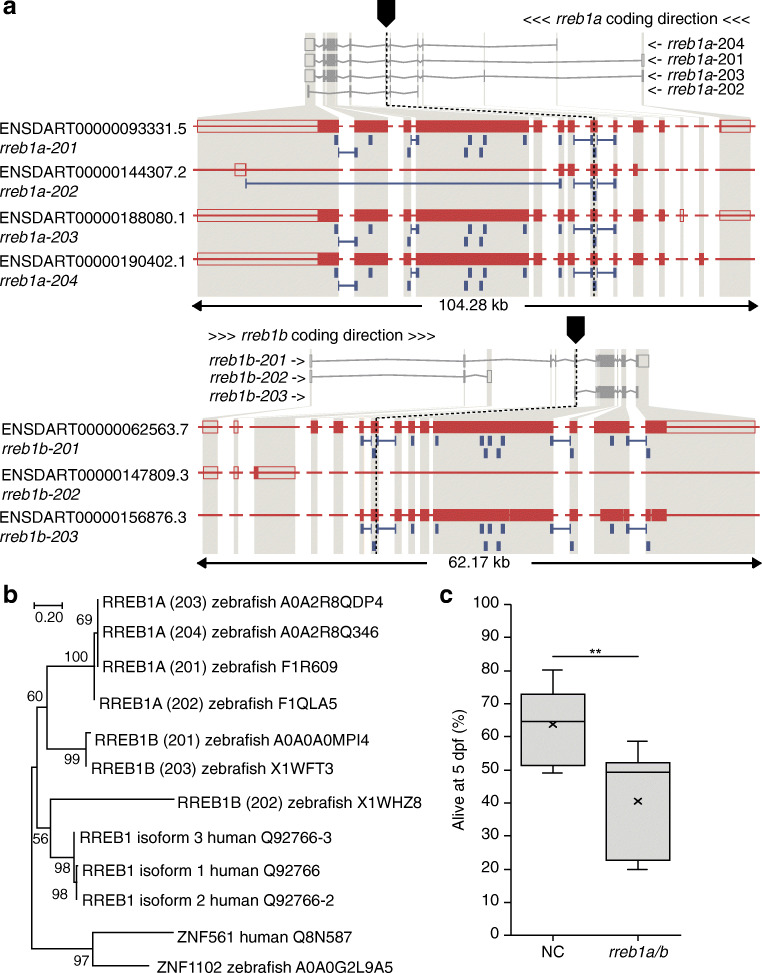
Generation of the *rreb1* loss-of-function zebrafish model. (**a**) Genomic structure of the zebrafish orthologues (*rreb1a* and *rreb1b*) of human *RREB1* and the sites targeted by CRISPR/Cas9 in each gene. (**b**) Phylogenetic tree of human and zebrafish RREB1 proteins. Numbers on nodes are bootstrap values; the uniprot accession numbers of the sequences used are given after the species name. (**c**) Box and whisker plot of the percentage of embryonic/larval survival from day 1 to day 5 post fertilisation for the control group (NC) and *rreb1a/b* crispants based on data from six independent experiments (the number of larvae per experiment ranged from 188 to 308 24 h after microinjection; 1806 larvae in total). **p<0.01 (paired Student’s *t* test)

As the *RREB1* locus is associated with altered diabetes risk and beta cell-related traits in humans, we first investigated the impact of loss of *RREB1* at an organismal level. The two zebrafish orthologues of the human *RREB1* gene—*rreb1a* and *rreb1b*—were targeted together in zebrafish (Fig. 1a) at the single-cell stage using CRISPR/Cas9 [23]. As the gene structures of *rreb1a* and *rreb1b* are very similar (Fig. 1a) and their amino acid sequences are more similar to each other than either is to the human RREB1 protein (Fig. 1b), the genes are likely to be remnants of the whole genome duplication in teleost fish [54]. To avoid compensatory effects between the two paralogues, we targeted all relevant transcripts of both genes (Fig. 1a; see Methods, CRISPR/Cas9 genome editing in zebrafish embryos for a detailed consideration of the target sites). Survival to 5 dpf was lower in *rreb1a*/*b* crispants than in the control group (Fig. 1c), but comparable to the survival of crispants for other cardiometabolic candidate genes.

**Fig. 2:**
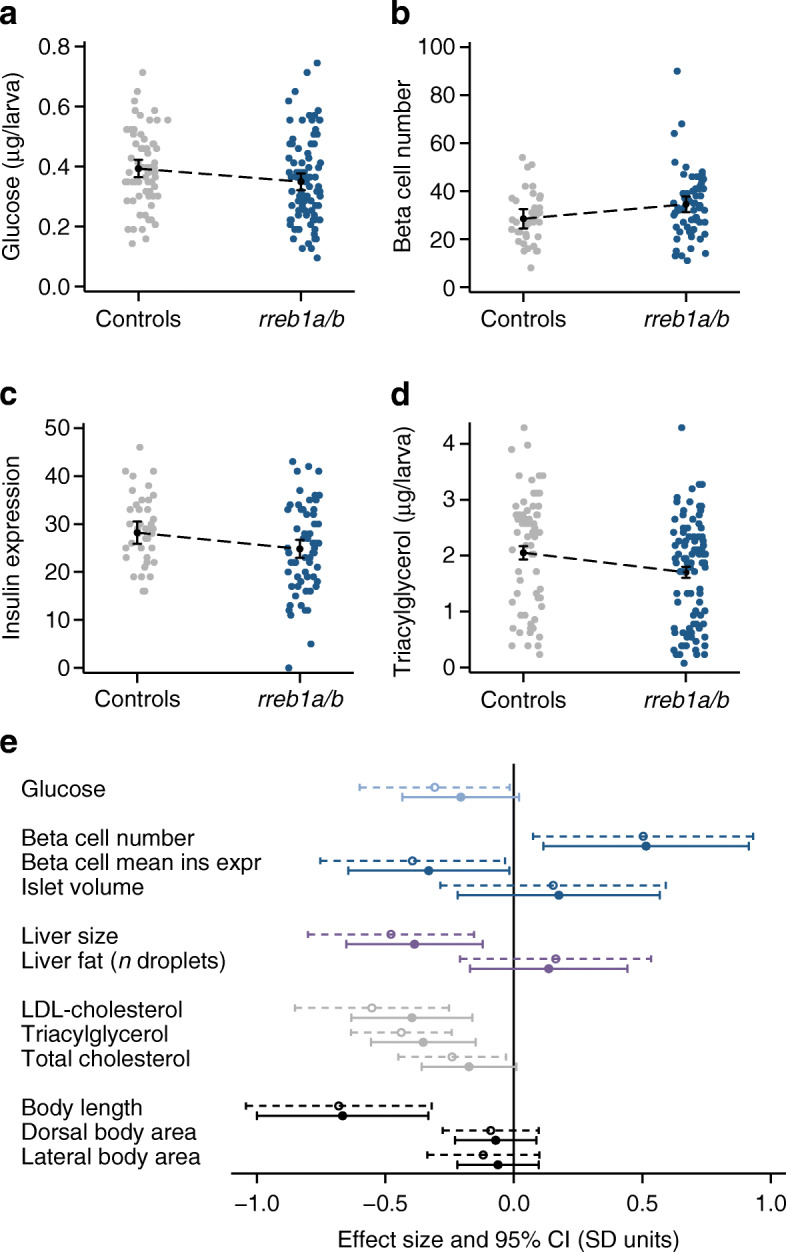
Effect of CRISPR/Cas9-induced mutations in zebrafish *rreb1a* and *rreb1b* on diabetes-related traits. (**a**–**d**) Individual-level data and margin plots for effects of mutations in *rreb1a/b* and *kita* (vs sibling controls targeted only at *kita*) on key traits with significant differences between the two groups, analysed using multiple linear regression analysis (*p*<0.05): (**a**) glucose levels, (**b**) beta cell number, (**c**) beta cell mean insulin expression (arbitrary units) and (**d**) triacylglycerol levels. Effects were adjusted for experiment, tank and time of day. Effects on glucose and triacylglycerol levels were additionally adjusted for imaging (yes/no) and for sample position and run. (**e**) Forest plot showing effect sizes and 95% CIs from multiple linear regression analysis for 10-day-old CRISPR/Cas9 founders with mutations in *rreb1a/b* and *kita* vs controls targeted only at *kita*. Dashed 95% CIs reflect results for crispants vs sibling controls only; solid 95% CIs show results including 536 additional controls from other experiments performed the same way. Adjustments are as described for Fig. 2a–d. Dorsal and lateral body area were additionally adjusted for length

Using image-based quantification of pancreatic beta cell and hepatic traits in 10 dpf transgenic larvae expressing H2B-mCherry under the control of the insulin promoter [18], crispants had more pancreatic beta cells with a lower mean signal from the nuclear reporter of insulin expression (Fig. 2). Crispants were also shorter and had lower glucose, LDL-cholesterol, triacylglycerol and total cholesterol levels and a smaller liver (Fig. 2). While not revealing the causal path by which mutations in *rreb1a/b* affect these traits, these results do support the pancreatic beta cell as a key tissue affected by loss of *rreb1a/b* in vivo. Effects of mutations in *rreb1a/b* on glucose should ideally be adjusted for size, but doing so is likely to result in biased estimates. Hence, future studies with blood samples obtained from older fish are required to confirm or refute the effect of mutations in *rreb1a/b* on glucose.

### RREB1 deficiency reduces *INS* expression and cellular insulin content in human EndoC-βH1 cells

**Fig. 3:**
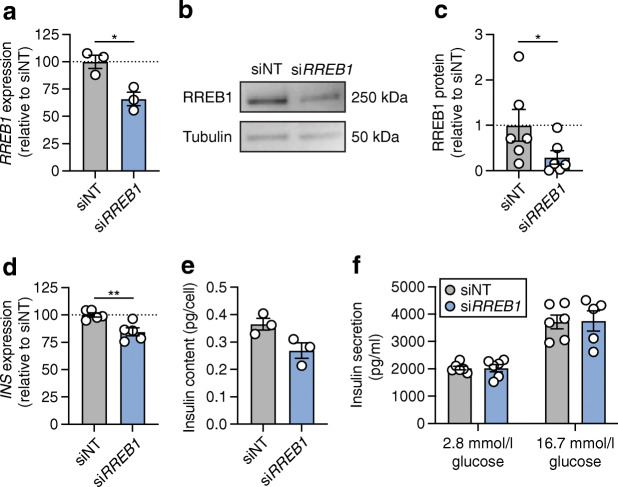
Partial loss of RREB1 reduces cellular insulin content in mature beta cells. (**a**) *RREB1* gene expression (normalised to the housekeeping genes *TBP* and *PPIA* and expressed as % of siNT) in siNT control and si*RREB1* knockdown EndoC-βH1 cells (*n*=3). (**b**) Representative western blot of RREB1 (250 kDa) and tubulin (50 kDa) in siNT and si*RREB1* EndoC-βH1 cells. (**c**) Quantification of RREB1 protein expression following siRNA knockdown in EndoC-βH1 cells (*n*=6). (**d**) *INS* gene expression (normalised to the housekeeping genes *TBP* and *PPIA* and expressed as % of siNT) in siNT and si*RREB1* EndoC-βH1 cells (*n*=5). (**e**) Cellular insulin content (pg/cell) (*n*=5) and (**f**) glucose-stimulated insulin secretion (pg/ml) (*n*=6; *n*=5 for si*RREB1* 16.7 mmol/l glucose) measured in siNT and si*RREB1* EndoC-βH1 cells. Data are presented as means±SEM. *p<0.05, **p<0.01 (unpaired *t* test)

Having established the effects of *rreb1* loss at an organismal level on beta cell insulin expression, we wanted to determine whether changes in *RREB1* expression and/or RREB1 activity alter human beta cell function. To assess the role of RREB1 in mature beta cells, we performed siRNA knockdown of *RREB1* in human EndoC-βH1 cells and assessed glucose-stimulated insulin secretion. Transfection of siRNAs in EndoC-βH1 cells reduced *RREB1* transcript levels by 34±9% and protein levels by 70±20% (Fig. 3a–c). *RREB1* knockdown decreased *INS* transcript levels by 16±4% (Fig. 3d) and cellular insulin content by 32±11% (Fig. 3e) compared with siNT controls. There was no effect on basal (2.8 mmol/l) or glucose-stimulated (16.7 mmol/l) insulin secretion following *RREB1* knockdown (Fig. 3f).

**Fig. 4:**
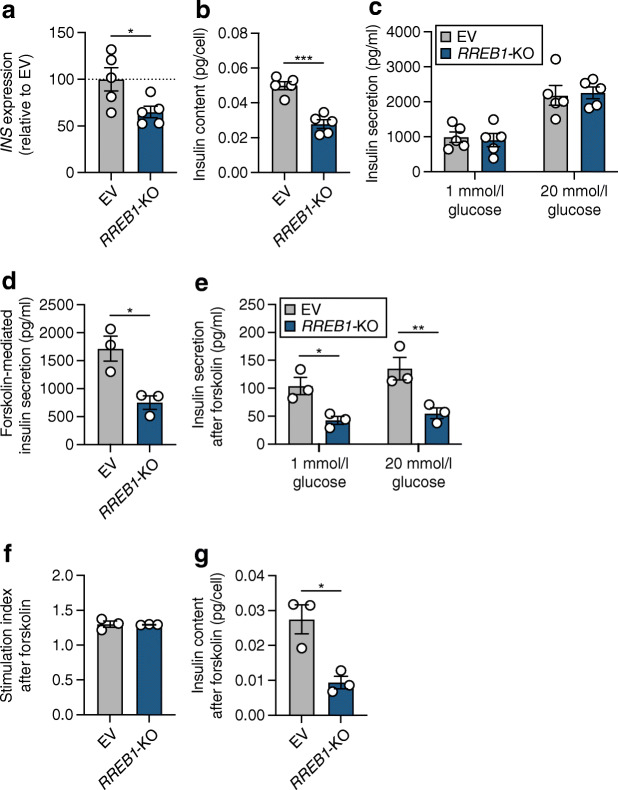
*RREB1* knockout reduces cellular insulin content in mature beta cells. (**a**–**c**) *RREB1* knockout (*RREB1*-KO) EndoC-βH1 cells were assessed for (**a**) *INS* gene expression (% of control cells [EV], normalised to housekeeping genes *TBP* and *PPIA*), (**b**) cellular insulin content (pg/cell) and (**c**) glucose-stimulated insulin secretion (pg/ml) (*n*=5). (**d**) Forskolin-mediated insulin secretion at 20 mmol/l glucose (pg/ml) (*n*=3). (**e**–**g**) After stimulation with forskolin, (**e**) glucose-stimulated insulin secretion (pg/ml), (**f**) the stimulation index and (**g**) insulin content (pg/cell) were measured (*n*=3). Data are presented as means±SEM. *p<0.05, **p<0.01, p***<0.001 (**a**, **b**, **d**, unpaired *t* test; **c**, **e**, two-way ANOVA followed by Sidak’s multiple comparisons test)

As we previously identified phenotypic differences between transient and long-term loss of function in the EndoC-βH1 model [36], we next used CRISPR/Cas9 to generate pooled knockout *RREB1* EndoC-βH1 cells (*RREB1*-KO). To control for the genome editing pipeline, empty vector (EV) control cells were generated that express Cas9 protein without sgRNAs to target the genome. Four sgRNAs targeting exons 4, 5, 8 and 12 of the protein-coding sequence of *RREB1* were used to generate *RREB1*-KO EndoC-βH1 cells (ESM Fig. 3a). After puromycin treatment, selected EndoC-βH1 cells represent a heterogeneous pool of cells that have no edits, an indel (insertion or deletion) resulting from binding of a single sgRNA, or a larger deletion from cutting of two or more sgRNAs. PCR analysis of genomic DNA from EV and *RREB1*-KO cells revealed a specific amplicon in *RREB1*-KO cells that would result only in cells with a deletion between exon 4 and exon 12 (ESM Fig. 3b). Sanger sequencing followed by TIDE analysis [37] of PCR products surrounding the cut sites at exons 4, 5, 8 and 12 revealed an editing efficiency of 2–24% (ESM Fig. 3c). *RREB1*-KO cells had a near complete loss of RREB1 protein compared with the parental and EV cells (ESM Fig. 3d), without altering the overall growth rate (ESM Fig. 3e). CRISPR/Cas9-mediated loss of RREB1 in EndoC-βH1 cells resulted in a 35±14% reduction in *INS* expression (Fig. 4a) and a 44±7% reduction in cellular insulin content (Fig. 4b) compared with EV cells. Of note, insulin content was decreased tenfold in EV control cells (Fig. 4b) compared with siNT (Fig. 3e), which is likely to be an artefact of the genome editing pipeline and highlights the importance of including an EV control. Similar to transient knockdown, insulin secretion from *RREB1*-KO EndoC-βH1 cells was not significantly different from that in control EV cells at basal or high glucose levels (Fig. 4c). Taken together, loss of *RREB1* in human beta cells reduces cellular insulin content but does not affect glucose-stimulated insulin secretion.

To assess the effect of lower insulin availability in *RREB1*-KO beta cells under conditions of prolonged insulin demand, cells were stimulated with 20 mmol/l glucose in combination with the cAMP-elevating agent forskolin (10 μmol/l) for 30 min prior to evaluation of glucose-stimulated insulin secretion. *RREB1*-KO EndoC-βH1 cells secreted less insulin in response to forskolin stimulation than control cells (Fig. 4d). Assessment of insulin release after forskolin-mediated docked granule depletion showed significantly reduced insulin secretion in response to glucose stimulation in RREB1-deficient EndoC-βH1 cells (Fig. 4e). While the response to glucose after the forskolin challenge was blunted in both *RREB1*-KO and control EndoC-βH1 cells (stimulation index: *RREB1*-KO 1.3±0.08; EV 1.3±0.003), *RREB1*-depleted cells did not recover as well as control EndoC-βH1 cells (*p=*0.06) (Fig. 4f). After forskolin treatment, the insulin content was reduced in *RREB1*-KO EndoC-βH1 cells (Fig. 4g), suggesting that loss of RREB1 negatively impacts insulin secretion during periods of prolonged demand.

### RREB1 is a novel transcriptional activator and repressor in mature beta cells

As RREB1 is a transcription factor, we next performed transcriptomic analysis in EndoC-βH1 cells following siRNA-mediated knockdown and CRISPR/Cas9 knockout. In total, 2144 DEGs (*q*<0.01) were detected between siNT- and si*RREB1*-treated samples, with slightly more upregulated genes (56%) in the RREB1-depleted cells (ESM Table 7). Approximately half of the DEGs (55% and 56% of upregulated and downregulated genes, respectively) corresponded to predicted RREB1 target genes identified in the JASPAR and TRANSFAC databases [55, 56]. Enriched biological terms and pathways among all upregulated DEGs included processes associated with neurons, such as ‘nervous system development’, ‘neuronal system’, ‘synaptic signalling’ and ‘axon guidance’, which is likely to reflect the phenotypic and transcriptomic similarities between neurons and beta cells [57]. In addition, terms relating to exocytotic processes, such as ‘regulation of exocytosis’, ‘synaptic vesicle exocytosis’ and ‘transmission across chemical synapses’, were also enriched in upregulated DEGs, consistent with the role of RREB1-regulated genes in insulin secretion.

Differential gene expression analysis identified 2604 DEGs between *RREB1*-KO and wild-type EV EndoC-βH1 cells, with more than half (66%) being upregulated as a consequence of RREB1 loss (ESM Table 8). There was a striking overlap in the DEGs (930 out of 2144) shared between the *RREB1* knockdown and knockout EndoC-βH1 cell models. The two *RREB1*-deficient EndoC-βH1 models shared 736 upregulated genes and 194 downregulated genes. *RREB1* gene expression was elevated in *RREB1*-KO cells compared with wild-type EV cells (*p*_adj_*=*2.84×10^−21^, log_2_FC=0.7444). However, this increased expression was the result of non-targeting of exons in the 5′-UTR (*p*_adj_*<*0.001) by the four sgRNAs and is consistent with genetic compensation for loss of RREB1. Other upregulated genes included transcripts involved in insulin secretion and processing (*CHGB*, *SNAP25*, *SCG2*), transcripts encoding voltage-sensitive Ca^2+^ channel subunits (*CACNA1B*, *CACNA1C*, *CACNA1D*, *CACNA1E*) and transcripts involved in cell-to-cell communication (*GJD2*, *NCAM1*, *PTPRN*), suggesting a potential compensatory effect for the reduced insulin content (ESM Table 8). Accordingly, upregulated DEGs were enriched for biological terms related to exocytosis and insulin secretion (ESM Table 9). Consistent with growth rate data (ESM Fig. 3e), gene ontology did not identify enrichment of terms relating to ‘cell cycle’ or ‘proliferation’. Expression of *NEUROD1*, which encodes a well-established regulator of the *INS* gene [58] and endocrine cell development [59], was significantly downregulated in *RREB1*-KO cells compared with EV EndoC-βH1 cells, suggesting that RREB1 may regulate genes involved in endocrine cell differentiation.

### RREB1 loss of function during in vitro differentiation affects endocrine progenitor development

To address the role of RREB1 during endocrine cell differentiation, we generated multiple isogenic *RREB1*^WT/WT^ and *RREB1*^KO/KO^ hiPSC cell lines. Four independent *RREB1*^KO/KO^ cell lines were generated using two sgRNAs that target sequences either close to the start codon (exon 4) or in a distal exon (exon 10). Both sgRNAs are located in genomic regions that are common to all protein-coding *RREB1* transcripts and generated an ~50 kb deletion (ESM Fig. 4a). As sequencing of the SB Ad3.1 hiPSC cell line revealed heterozygosity for the common type 2 diabetes-associated *RREB1* variant rs9379084 (c.3511G>A, p.Asp1171Asn), *RREB1*^WT/WT^ cell lines were genetically edited to be homozygous for the major allele at rs9379084, which is associated with a higher risk of type 2 diabetes (c.3511G, p.Asp1171Asp) (ESM Fig. 4a). Quantification of RREB1 protein showed no difference in expression levels between the three edited *RREB1*^WT/WT^ clones and an unedited parental SB Ad3.1 (p.Asp1171Asn) hiPSC cell line (ESM Fig. 4b, c). RREB1 protein was not detectable in any of the four *RREB1*^KO/KO^ hiPSC cell lines by western blotting and immunofluorescent staining (ESM Fig. 4b–d). All gene-edited *RREB1*^KO/KO^ hiPSC cell lines expressed pluripotency markers (octamer-binding transcription factor 4 [OCT4], sex-determining region Y-box 2 [SOX2], NANOG and stage-specific embryonic antigen 4 [SSEA4]) (ESM Fig. 4e) and showed no change in proliferation (ESM Fig. 4f). Genome-engineered hiPSC cell lines also had typical hiPSC morphology, a diploid karyotype and none of the ten most frequently detected coding mutations in *TP53* as a result of the genome editing process (ESM Table 5).

**Fig. 5:**
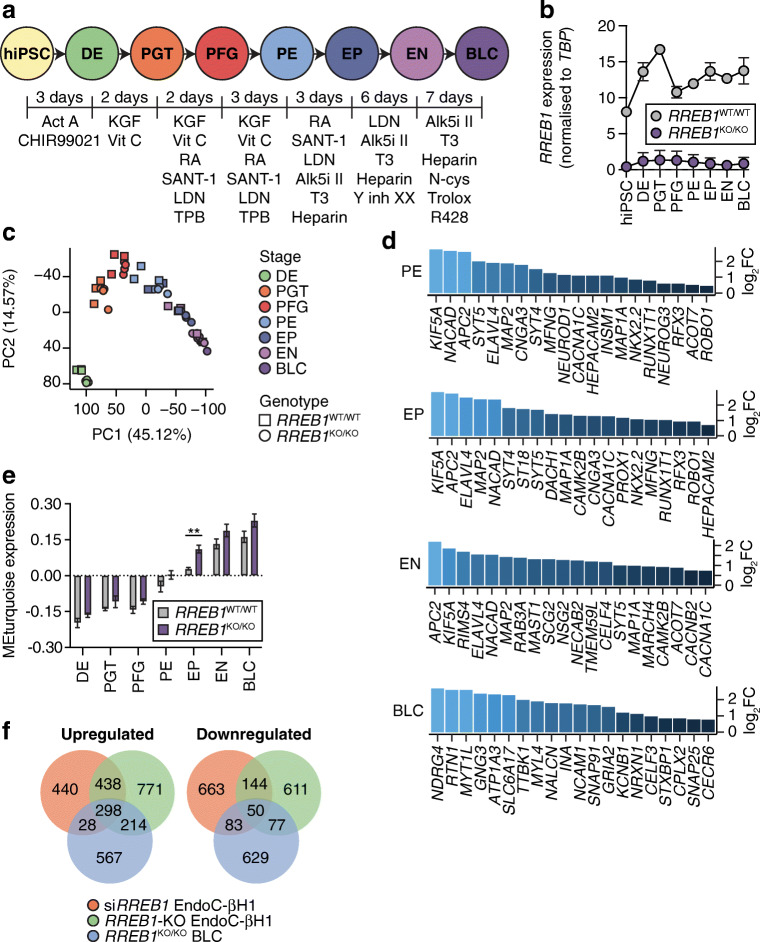
Transcriptomic analysis reveals altered expression of pro-endocrine genes following loss of *RREB1* in human beta cells. (**a**) Schematic of the in vitro differentiation protocol stages: definitive endoderm (DE) cells, primitive gut tube (PGT) cells, posterior foregut (PFG) cells, pancreatic endoderm (PE) cells, endocrine progenitor (EP) cells, endocrine (EN) cells and BLCs. Growth factors and small molecules (listed underneath each stage) were added for the indicated amounts of time. (**b**) *RREB1* expression in *RREB1*^WT/WT^ (*n*=3) and *RREB1*^KO/KO^ (*n*=4) hiPSC cell lines during in vitro differentiation towards BLCs. (**c**) The first two principal components (PC1, PC2) were calculated using normalised gene counts of *RREB1*^KO/KO^ (circles; *n*=4) and *RREB1*^WT/WT^ (squares; *n*=3) cell lines for all seven stages of in vitro beta cell differentiation. (**d**) Differential expression of endocrine cell genes in PE cells, EP cells, EN cells and BLCs. (**e**) Analysis of modules of co-expressed genes using WGCNA: bar plot showing module epigengene (ME) expression of the module enriched for endocrine progenitor and endocrine genes. (**f**) Venn diagrams of the overlap of DEGs between si*RREB1* knockdown EndoC-βH1 cells, *RREB1*-KO EndoC-βH1 cells and hiPSC-derived *RREB1*^KO/KO^ BLCs. Data are presented as means±SEM. **p<0.01 (unpaired *t* test). Act A, activin A; Alk5i II, ALK5 inhibitor II; CHIR99021, GSK-3 inhibitor; KGF, keratinocyte growth factor; LDN, LDN193189 BMP type 1 receptor inhibitor; N-cys, N-acetyl cysteine; R428, AXL inhibitor; RA, retinoic acid; SANT-1, hedgehog signalling inhibitor; T3, triiodothyronine; TPB, PKC activator; Trolox, vitamin E; Y inh XX, gamma secretase inhibitor; Vit C, vitamin C

To model endocrine pancreas development, we differentiated the *RREB1*^WT/WT^ and *RREB1*^KO/KO^ hiPSC cell lines along the endocrine lineage into BLCs [42] and performed transcriptomic analysis at all seven stages of in vitro differentiation (Fig. 5a). *RREB1* was expressed at all stages of beta cell differentiation in *RREB1*^WT/WT^ cells and its expression was significantly reduced in *RREB1*^KO/KO^ cells (Fig. 5b). Stage-specific marker expression revealed that *RREB1*^KO/KO^ and *RREB1*^WT/WT^ cells followed established endocrine development stages and generated BLCs characterised by co-expression of NKX6.1 and C-peptide (ESM Fig. 5a, b). Principal component analysis revealed that both *RREB1*^WT/WT^ and *RREB1*^KO/KO^ samples clustered by developmental stage in the expected pattern, with more variability observed in the later stages (Fig. 5c). Differential expression analysis at each differentiation stage revealed that loss of RREB1 resulted in a total of 5476 DEGs between *RREB1*^WT/WT^ and *RREB1*^KO/KO^ cells, of which 159 were common to all developmental stages (ESM Table 10). The majority of DEGs were upregulated in the *RREB1*^KO/KO^ cell lines (63±5%) and were found at the endocrine progenitor stage. Upregulated DEGs in *RREB1*^KO/KO^ hiPSC-derived pancreatic endoderm, endocrine progenitor and endocrine cells were enriched for genes involved in the ‘regulation of gene expression in endocrine-committed (NEUROG3+) progenitor cells’, ‘insulin secretion’ and ‘regulation of insulin secretion’, respectively (ESM Table 11). Interestingly, transcript expression of the endocrine progenitor marker *NEUROG3* was significantly higher in *RREB1*^KO/KO^ hiPSC-derived endocrine progenitor cells (ESM Fig. 5c), suggesting accelerated differentiation towards the endocrine lineage.

Using stage-specific markers identified in human fetal pancreases [60], hypergeometric enrichment analyses revealed an enrichment of endocrine progenitor markers (*NEUROG3*, *NEUROD1*, *NKX2.2*, *RFX3*, *CACNA1C*) among genes upregulated in *RREB1*^KO/KO^ cell lines in pancreatic endoderm (*q*=4.0×10^−83^), endocrine precursor (*q*=6.3×10^−104^) and endocrine (*q*=5.5×10^−43^) cells (Fig. 5d). Among genes upregulated in *RREB1*^KO/KO^ BLCs, there was an enrichment of genes implicated in insulin exocytosis (*SNAP25*, *STXBP1*, *NRXN1*) (*q*=1.6×10^−35^) (Fig. 5d). Downregulated DEGs were enriched in early and late pancreatic progenitors (*q*=3.2×10^−28^ and *q*=7.2×10^−24^, respectively); these included two acinar cell markers (*CPA2* and *NR5A2*), the multipotent pancreatic progenitor transcription factor *HNF1B* [61, 62] and members of the Notch signalling (*NOTCH1*, *NOTCH2*, *JAG1*) and EGF and FGF (*ERBB3*, *FGFR2*) pathways. To identify co-expressed genes that may be regulated by RREB1, WGCNA [49, 50] was performed. The module eigengene turquoise (MEturquoise), enriched for endocrine progenitor and endocrine genes, showed significant expression differences between *RREB1*^WT/WT^ and *RREB1*^KO/KO^ cells (Fig. 5e; ESM Table 12). Interestingly, a subset of significantly upregulated and downregulated genes was shared among the si*RREB1* EndoC-βH1 cells, *RREB1*-KO EndoC-βH1 cells and hiPSC-derived *RREB1*^KO/KO^ BLCs (Fig. 5f), suggesting a common RREB1 regulatory network between developing and mature beta cells.

### Loss of RREB1 increases RFX motif activity during endocrine cell differentiation and in mature beta cells

**Fig. 6:**
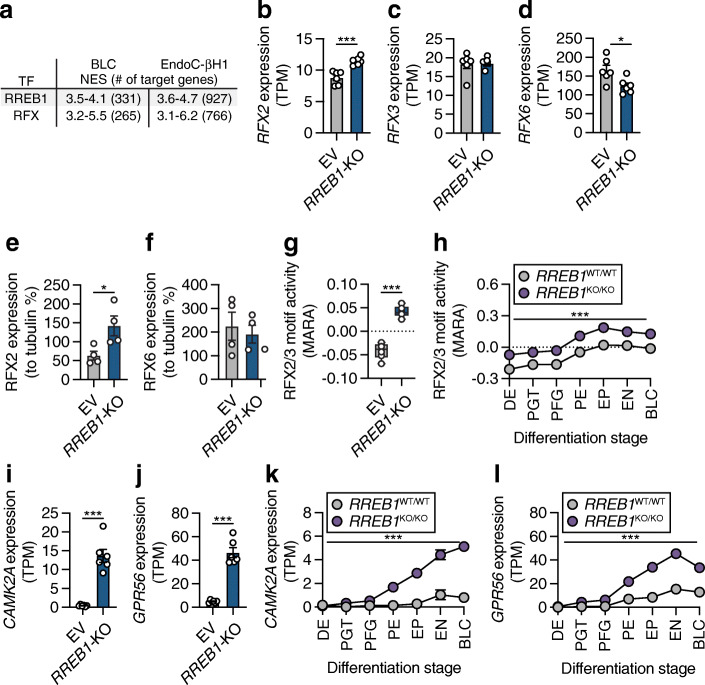
RREB1 deficiency affects RFX motif activity. (**a**) Most common transcription factor (TF) motifs in hiPSC-derived BLCs and EndoC-βH1 cells of 9713 position weight matrices and 1120 ENCODE ChIP-Seq tracks (centred 10kb around a transcription start site) tested. NES, normalised enrichment score with cut-off set to >3 (corresponding to a false discovery rate of 3–9%); # of target genes, number of target genes for the TF motif with the highest NES. (**b**–**d**) Expression of (**b**) *RFX2*, (**c**) *RFX3* and (**d**) *RFX6* mRNA in transcripts per millions (TPM) in EV and *RREB1-*KO EndoC-βH1 cells (*n*=6). (**e**, **f**) Protein quantification of (**e**) RFX2 and (**f**) RFX6 in RREB1-deficient cells (*n*=4). (**g**, **h**) RFX2/3 motif activities in (**g**) EV and *RREB1-*KO EndoC-βH1 cells (*n*=6) and (**h**) *RREB1*^WT/WT^ (*n*=3) and *RREB1*^KO/KO^ (*n*=4) cells during hiPSC differentiation to BLCs calculated using MARA. (**i**, **j**) Expression of the RFX2/3 target genes (**i**) *CAMK2A* and (**j**) *GPR56* in EV and *RREB1-*KO EndoC-βH1 cells (*n*=6). (**k**, **l**) Expression of the RFX2/3 target genes (**k**) *CAMK2A* and (**l**) *GPR56* in *RREB1*^WT/WT^ (*n*=3) and *RREB1*^KO/KO^ (*n*=4) cells during in vitro differentiation to BLCs. Data are presented as means±SEM. *p<0.05, ***p<0.001 (unpaired *t* test)

Computational prediction of upstream regulators of the DEGs in hiPSC-derived BLCs and EndoC-βH1 cells using iRegulon [51] highlighted RREB1, as well as the RFX transcription factor family (Fig. 6a). The RFX family comprises eight members and is characterised by a highly conserved DNA-binding domain [63, 64]. Loss of *RREB1* significantly increased *RFX2* expression (*q*=6.47×10^−4^, log_2_FC=0.3629) and decreased *RFX6* expression (*q*=3.06×10^−8^, log_2_FC=−0.4188) in EndoC-βH1 cells, while expression of *RFX3* was unchanged (Fig. 6b–d). Interestingly, while RFX2 protein expression was markedly increased in *RREB1*-KO EndoC-βH1 cells (22.26±0.10-fold, *p*=0.0123), loss of RREB1 did not affect RFX6 protein expression in mature beta cells (Fig. 6e,f; ESM Fig. 6a).

MARA, a further approach to predict genome-wide regulatory interactions that underlay gene expression variation across *RREB1*-deficient cells, predicted RFX2 and RFX3 as key transcription factors driving differential gene expression across *RREB1*-KO cells in EndoC-βH1 cells (RFX2/3 *Z*=8.45) (Fig. 6g) and during beta cell differentiation (RFX2/3 *Z*=12.43) (Fig. 6h). RFX2/3 target genes *CAMK2A* [65] and *ADGRG1* (*GPR56*) [66] were among the DEGs showing the strongest upregulation in *RREB1*-KO EndoC-βH1 cells and across all seven in vitro differentiation stages (Fig. 6i–l). Taken together, the transcriptomic analysis revealed *RFX* family members as potential targets of RREB1 in beta cells.

**Fig. 7:**
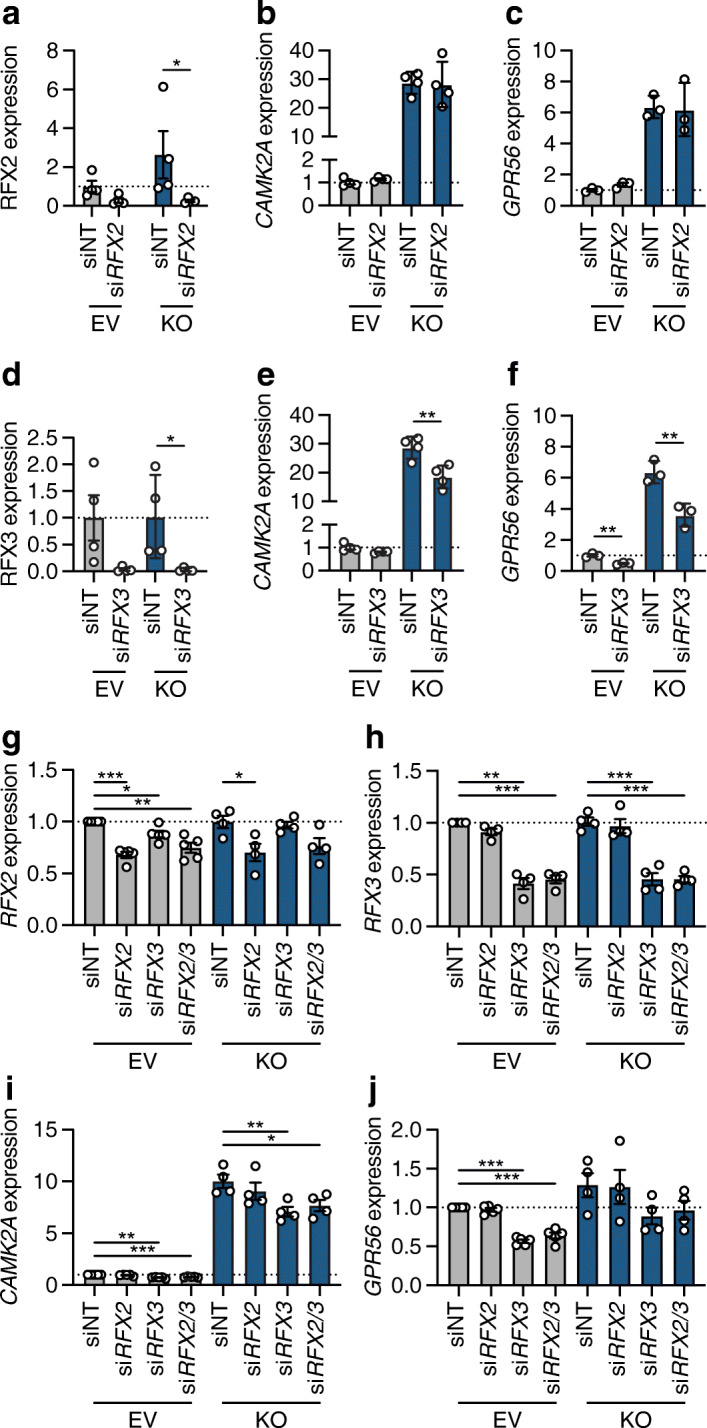
RREB1 deficiency in EndoC-βH1 cells alters gene expression of *RFX* family members. (**a**) RFX2 protein quantification (normalised to tubulin and relative to EV siNT) in EV (*n*=4) and *RREB1*-KO (*n*=4) EndoC-βH1 cells following siNT and si*RFX2* transfection. (**b**, **c**) Gene expression of (**b**) *CAMK2A* (*n*=4) and (**c**) *GPR56* (*n*=3) (normalised to the housekeeping genes *TBP* and *PPIA* and relative to siNT) in EV and *RREB1*-KO EndoC-βH1 cells following siRNA-mediated depletion of *RFX2*. (**d**) RFX3 protein quantification (normalised to tubulin and relative to EV siNT) in EV (*n*=4) and *RREB1*-KO (*n*=4) EndoC-βH1 cells following siNT and si*RFX3* transfection. (**e**, **f**) Gene expression of (**e**) *CAMK2A* (*n*=4) and (**f**) *GPR56* (*n*=3) (normalised to the housekeeping genes *TBP* and *PPIA* and relative to siNT) in EV and *RREB1*-KO EndoC-βH1 cells following siRNA-mediated deletion of *RFX3*. (**g**, **h**) Gene expression of (**g**) *RFX2* (EV, *n*=5; *RREB1*-KO, *n*=4) and (**h**) *RFX3* (*n*=4) (normalised to the housekeeping gene *TBP* and relative to siNT) in EV and *RREB1*-KO EndoC-βH1 cells following siRNA-mediated knockdown of *RFX2*, *RFX3* or *RFX2* and *RFX3*. (**i**, **j**) Gene expression of (**i**) *CAMK2A* and (**j**) *GPR56* (normalised to the housekeeping gene *TBP* and relative to siNT) in EV (*n*=5) and *RREB1*-KO (*n*=4) EndoC-βH1 cells following single or dual knockdown of *RFX2* and *RFX3*. Data are presented as means±SEM. *p<0.05, **p<0.01, ***p<0.001 (**a**–**f**, unpaired *t* test; **g**–**j**, unpaired *t* test with Welch’s correction)

Our in silico approaches were unable to distinguish between RFX2 and RFX3 owing to their similar binding motifs. Thus, we next used RNA interference-mediated inhibition of both *RFX2* and *RFX3* to determine if the changes in gene expression following RREB1 loss could be mirrored by modulating RFX proteins in beta cells. Loss of RFX2 protein following RNA interference (Fig. 7a; ESM Fig. 6b) did not impact expression of the target genes *CAMK2A* and *GPR56* (Fig. 7b, c). RNA interference-driven reductions in RFX3 protein levels (Fig. 7d; ESM Fig. 6c) partially rescued increased *CAMK2A* and *GPR56* expression in *RREB1*-KO beta cells (Fig. 7e, f). As RFX2/3 share a binding motif, we next investigated whether there was functional redundancy by performing dual knockdown studies. Knockdown of *RFX3* alone or in combination with *RFX2* (Fig. 7g, h) was sufficient to decrease expression of *CAMK2A* (Fig. 7i) and *GPR56* (Fig. 7j), supporting a role for RFX3 as a transcriptional regulator affected by loss of RREB1 in mature beta cells.

### Carriers of type 2 diabetes risk alleles in RREB1 have altered beta cell function

**Fig. 8:**
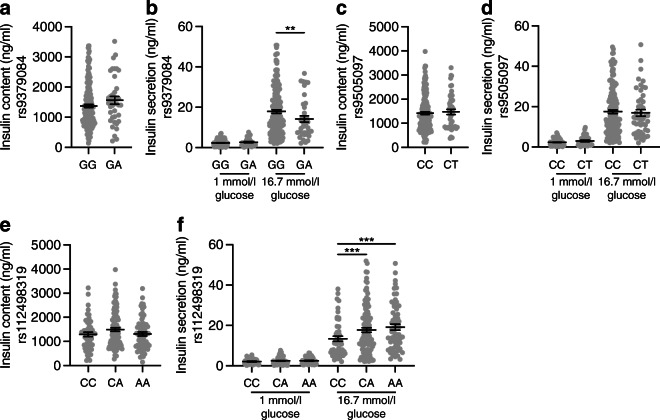
Genetic variation at the *RREB1* locus influences human beta cell function. Insulin content (**a**, **c**, **e**) and glucose-stimulated insulin secretion (**b**, **d**, **f**) in human donor islets from carriers of *RREB1* variants: (**a**, **b**) rs9379084 (GG, *n*=180; GA, *n*=38), (**c**, **d**) rs9505097 (CC, *n*=168; CT, *n*=219) and (**e**, **f**) rs112498319 (CC, *n*=50; CA, *n*=109; CC, *n*=50). Data are presented as means±SEM. **p<0.01, ***p<0.001 (one-way ANOVA)

RREB1 loss of function in a human beta cell model negatively impacted insulin content and secretion. To determine whether all three independent signals at the *RREB1* locus influence pancreatic islet function, we quantified glucose-stimulated insulin secretion in ex vivo human islets stratified by genotype. For the causal coding variant (rs9379084), glucose-stimulated insulin secretion was paradoxically higher in carriers of the type 2 diabetes risk allele (G; p.Asp1171) and there was no statistically significant difference in insulin content (Fig. 8a, b). Neither of the index variants at the two regulatory signals (rs9505097 and rs112498319) influenced insulin content (Fig. 8c–e). However, carriers of the rs112498319 type 2 diabetes risk allele (C) showed lower mean glucose-stimulated insulin secretion levels (Fig. 8f). Together, these results support a role for RREB1 in human pancreatic islet function and suggest that at least two of the three signals at the locus alter islet cell function.

## Discussion

Our understanding of the genetic landscape of type 2 diabetes has increased substantially [2, 5, 67] and current efforts are focused on translating these genetic discoveries into disease mechanisms. Here, we characterised the role of the type 2 diabetes-associated gene *RREB1* in beta cell development and function. Our in vivo zebrafish model lacking *rreb1a* and *rreb1b* had reductions in beta cell insulin expression and increased beta cell numbers. Loss of RREB1 also reduced insulin gene expression and cellular insulin content in EndoC-βH1 cells, resulting in impaired glucose-stimulated insulin secretion under prolonged stimulation. Transcriptomic analysis identified RREB1 as a novel transcriptional activator and repressor in developing and mature human beta cells. Isolated human islets from carriers of the *RREB1* coding allele that is associated with lower diabetes risk (p.Asn1171) had lower glucose-stimulated insulin secretion. Taken together, our data are consistent with type 2 diabetes-protective alleles in RREB1 resulting in loss of function. The contradictory finding that carriers of the *RREB1* protective allele have lower insulin secretion levels but are protected from type 2 diabetes hints at potential additional functions of RREB1 in other diabetes-relevant tissues (e.g. insulin-responsive tissues).

Loss of RREB1 led to a significant increase in transcriptional activity of *RFX2* and *RFX3* in both the hiPSC-based developmental model and the mature EndoC-βH1 model. While RFX3 and RFX6 have been implicated in beta cell development, formation and function [68–73], a role for RFX2 has not yet been described. Human mapping of protein–protein interactions revealed that RFX6 physically interacts with RFX2 and RFX3 [74]; however, whether RFX2 and RFX3 form heterodimers in beta cells to cooperatively regulate gene expression is currently unknown. Loss of RREB1 in mature beta cells increased the expression of *RFX2* transcript and levels of RFX2, highlighting RREB1 as a transcriptional repressor of *RFX2* in mature beta cells and probably also during endocrine cell differentiation. A study aimed at the prediction of upstream transcriptional regulators of *RFX* genes using transcription factor binding profile analysis did not identify the RREB1 transcription factor binding site as being statistically over-represented in *RFX* promoters [64]. This suggests that RREB1 regulation of *RFX* expression is likely to be indirect. In addition, whether *RREB1* allele carriers have alterations in other pancreatic or intestinal endocrine cells, similar to mutations in *RFX6* [71], remains to be determined.

*GPR56* is one of the RFX target genes that was significantly upregulated in *RREB1*-deficient cell models. While *GPR56* is expressed at low levels in EndoC-βH1 cells, it is the most abundant G protein-coupled receptor transcript in mouse and human islets and its expression is reduced in several models of diabetes [75–77]. Treating mouse beta cells with the endogenous agonist of GPR56 increases intracellular Ca^2+^ and stimulates insulin secretion [78]. In addition to *GPR56* upregulation, *RREB1*-deficient human beta cells upregulated other genes involved in insulin secretion and insulin processing and genes encoding voltage-sensitive Ca^2+^ channel subunits. We hypothesise that the differential expression of these genes results from a compensatory mechanism for the reduction in insulin content.

One of the consistent phenotypes across the human and zebrafish models is the reduction in insulin expression. An important transcriptional activator of the *INS* gene is the transcription factor neuronal differentiation 1 (NEUROD1) [58], which co-occupies the *Ins1* and *Ins2* promoters with the C-terminal-binding protein 1 (CtBP)/RREB1 co-repressor complex in murine beta cells [79]. As such, it is tempting to hypothesise that RREB1 and NEUROD1 may also interact to transcriptionally regulate expression of the human *INS* gene.

In the absence of a validated assay to quantify zebrafish insulin levels, we used a transgenically expressed beta cell reporter with H2B-mCherry expression under the control of the insulin promoter. The downsides of this approach are that insulin promoter activity may not reflect the more physiologically relevant plasma insulin concentration and that transcriptional regulation and H2B-mCherry turnover may not reflect endogenous insulin expression. However, the integration of gene expression over a longer time frame, as happens with a reporter such as H2B-mCherry, could be considered advantageous, as it is less prone to short-term effects introduced by interindividual differences in, for example, feeding status during analysis. Importantly, the total fluorescence intensity of H2B-mCherry across segmented insulin-expressing nuclei correlated significantly (*R*^2^=0.18 and *p*<0.001) with *ins* expression in larvae (ESM Fig. 1), indicating that the reporter system generates a valid readout.

We targeted *rreb1a* and *rreb1b* in zebrafish using a global CRISPR/Cas9 approach. According to single-cell expression studies, these genes are expressed in multiple islet cell types of adult zebrafish [80] and in other tissues of larval-stage zebrafish [81]. Although directionally consistent results in human cellular beta cell models indicate a direct role for beta cells, we cannot formally conclude that the phenotypes observed in *rreb1a/b* crispants are mediated only by direct effects of altered *rreb1a/b* expression on beta cell function. Future studies of cell type-specific inactivation of *rreb1a/b* are required to confirm or refute a role for other cell types of relevance for type 2 diabetes.

Our study, in which we characterised an in vivo zebrafish model, two complementary *RREB1* knockout human cellular beta cell models, and ex vivo islet cell function in human carriers of *RREB1* alleles, strongly suggests a novel role for RREB1 in beta cell development and function through a transcriptional effect of RREB1 on endocrine cell-specific gene expression. Identification of RREB1 as a regulator of multiple genes of known importance in endocrine cell development and insulin secretion has important implications for the future evaluation of type 2 diabetes risk-associated variants, as they may exert their effects through modification of RREB1-binding sites in islet cells.

## Supplementary information

ESM 1 (PDF 1511 kb)

ESM 2 (XLSX 1002 kb)

ESM 3 (XLSX 1286 kb)

## Data Availability

The datasets generated and/or analysed during the current study are available from the European Genome-phenome Archive (https://ega-archive.org) under accession number EGAS00001006314. The script is available from the corresponding author on request.

## Abbreviations

BLC: Beta-like cell
DEG: Differentially expressed gene
dpf: Day(s) post fertilisation
EV: Empty vector
hiPSC: Human induced pluripotent stem cell
MARA: Motif activity response analysis
qPCR: Quantitative real-time PCR
RREB1: Ras-responsive element binding protein 1
*RREB1*-KO: Knockout EndoC-βH1 model
*RREB1*^KO/KO^: Knockout hiPSC cell line
sgRNA: Single guide RNA
TIDE: Tracking of Indels by DEcomposition
WGCNA: Weighted gene co-expression network analysis
