# Supplementary material for: Loss of RREB1 in pancreatic beta cells reduces cellular insulin content and affects endocrine cell gene expression
Source: Diabetologia. 2023 Jan 12;66(4):674–94. doi: 10.1007/s00125-022-05856-6 (PMC9947029; doi:10.1007/s00125-022-05856-6)
Supplement: Supplementary file 1 — (PDF 1511 kb) [file 125_2022_5856_MOESM1_ESM.pdf]

ESM Methods

Husbandry and transgenic lines

Eggs from *Tg(ins:Hsa.HIST1H2BJ-mCherry)*^{vu513} and *Tg(-2.8fabp10a:EGFP)*^{as3TG} positive zebrafish were targeted at the *kita* gene at the single cell stage to reduce pigmentation and were fed from 5 dpf to 9 dpf as described in section "*CRISPR/Cas9 in zebrafish embryos*". At 4 dpf, larvae were counted and distributed to six 1-litre tanks with 10 ml of filtered water per larva (40-60 larvae per tank). Three tanks were assigned to a 3% (w/v) glucose (Sigma-Aldrich) challenge and 3 tanks were controls (filtered water). Glucose treatment started at 5 dpf and larvae from all tanks were transferred into clean tanks with fresh water/glucose water daily, to reduce microbial growth in glucose water.

Imaging and image analysis of whole larvae and beta cell nuclei was carried out at 10 dpf as described in the "*Imaging of zebrafish larvae*" section with the following changes. Controls and glucose challenged larvae were imaged alternatingly. After imaging, larvae in 150 μl water were immediately collected into 1.5 ml vials containing 150 μl 2X DNA/RNA protection solution (Monarch Total RNA Miniprep Kit (New England Biolabs, USA)). Samples were stored at 4°C until the end of each imaging batch and then stored at -20°C until further processing.

RNA extraction of RNA > 200 nucleotides was carried out according to the standard protocol for RNA extraction from tissues or leukocytes using the Monarch Total RNA Miniprep Kit. After homogenizing larvae at max speed for 3 mins in 300 μl 1x DNA/RNA protection solution and three 1.4 mm acid washed zirconium beads (OPS diagnostics, USA) using a MiniG™ 1600 homogenizer (SPEX® SamplePrep, USA), the recommended on-bead DNase treatment was performed. RNA was eluted in 35 μl nuclease-free water. Residual genomic DNA was removed using the Turbo DNase Kit (ThermoFisher Scientific, USA) according to the manufacturer's protocol; 19-90 ng of this RNA were used for reverse transcription using the High-Capacity cDNA

Reverse Transcription Kit with RNase Inhibitor (Applied Biosystems™, ThermoFisher Scientific, USA) according to the manufacturer's protocol. cDNA was diluted 20x in nuclease-free water.

A reference sample reverse transcribed from a mix of the five most concentrated RNA samples was used to generate a 2x dilution series. A RT-negative sample generated from a mix of equal volumes from five samples that together resulted in 90 ng was made to confirm the absence of detectable genomic DNA. Real time qPCR was carried out in triplicate per sample, standard series, reverse transcription-negative sample and no template control sample using the PowerUp™ SYBR™ Green Master Mix on a StepOne Plus real-time PCR machine (Applied Biosystems™, ThermoFisher Scientific, USA). Reaction volumes were 10 μl; with 3 μl 20x diluted sample and primer concentrations between 250-500 nmol/l. Primer sequences are shown in **ESM Table 4**. The following two-step cycling protocol was used: 1x 50°C 2 mins, 1x 95°C 2 mins, 40x (95°C 3 secs, 63°C 30 secs), melt curve 60-95°C. Expression data was determined using the standard curves; expression per sample was normalized by the average expression of *actb1* and *eef1a1*.

ESM Tables

ESM Table 1: *RREB1* orthologues in Danio rerio (Ensembl release 105 - Dec 2021).

ESM Table 2: Gene IDs, CRISPOR scores and gRNA target sequences for zebrafish genes *rreb1a*, *rreb1b* and *kita*.

ESM Table 3: Sequences of primers used for fragment length analysis.

ESM Table 4: Sequences of primers used for qPCR based analysis of mutagenesis efficiency and gene expression in zebrafish larvae.

ESM Table 5: Ten most common coding mutations in *TP53* that were assessed in genome-edited hiPSC lines.

ESM Table 6: Composition of basal and complete differentiation media.

ESM Table 7: List of DEGs between si*RREB1* and siNT EndoC-βH1 cells.

ESM Table 8: List of DEGs between *RREB1*-KO and EV EndoC-βH1 cells.

ESM Table 9: Enriched biological terms and pathways among DEGs between *RREB1*-KO and EV EndoC-βH1 cells.

ESM Table 10: List of DEGs between $RREB1^{KO/KO}$ and $RREB1^{WT/WT}$ hiPSC during seven stages of *in vitro* beta cell differentiation.

ESM Table 11: Enriched biological terms and pathways among DEGs up-regulated in $RREB1^{KO/KO}$ lines at seven distinct stages of in vitro beta cell differentiation.

ESM Table 12: Module eigengenes identified in WGCNA for $RREB1^{KO/KO}$ and $RREB1^{WT/WT}$ lines during seven stages of in vitro beta cell differentiation.

ESM Figures

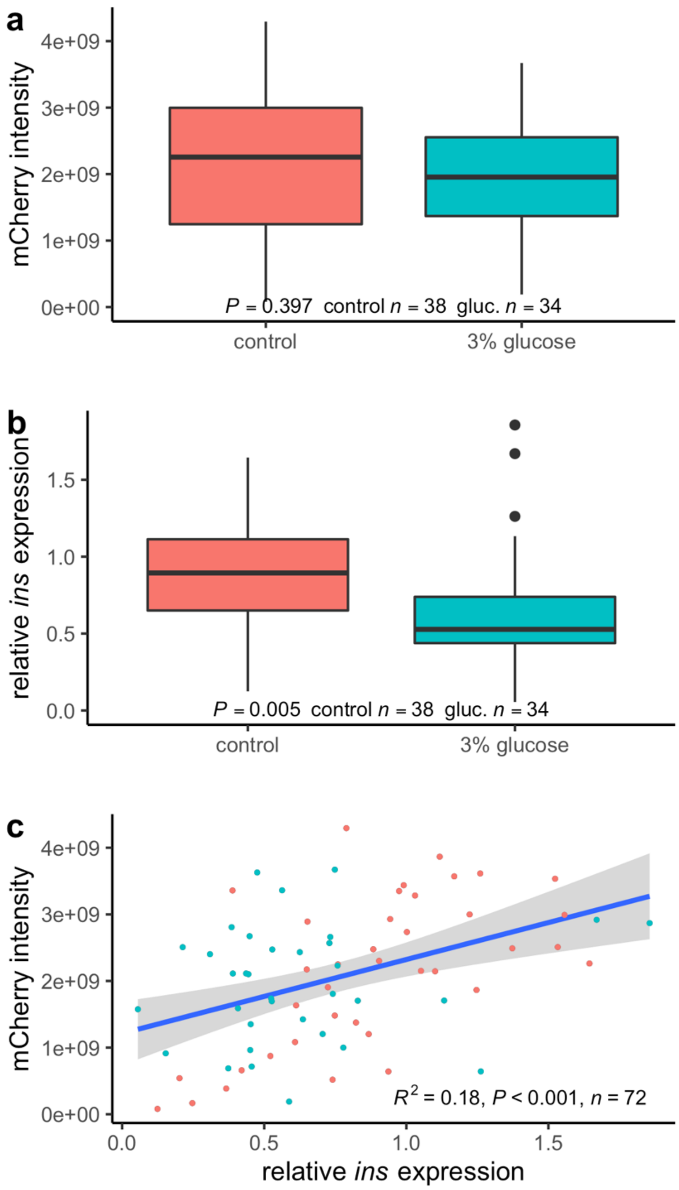

ESM Fig. 1: Validation of the *ins:H2B-mCherry* reporter in 10 dpf zebrafish larvae.
Red = control (normal water), green = 3% glucose (normal water with 3% glucose from 5 to 10 dpf); (**a**) H2B-mCherry expressed under the control of the *insulin* promoter was quantified through image-based analysis where mCherry intensity is the total fluorescence intensity across segmented insulin-expressing nuclei; (**b**) *ins* expression measured by qPCR in homogenates of single larvae, normalized to the average expression of *actb1* and *eef1a1*; (**c**) mCherry total fluorescence intensity across segmented insulin expressing nuclei as a function of the relative *ins* expression of the same sample quantified using qPCR; Statistics: (**a** and **b**) students t-test; (**c**) Pearson correlation. Data were analyzed using R [1] and the packages (tidyverse, ggpubr, ggtext and ggplot2).

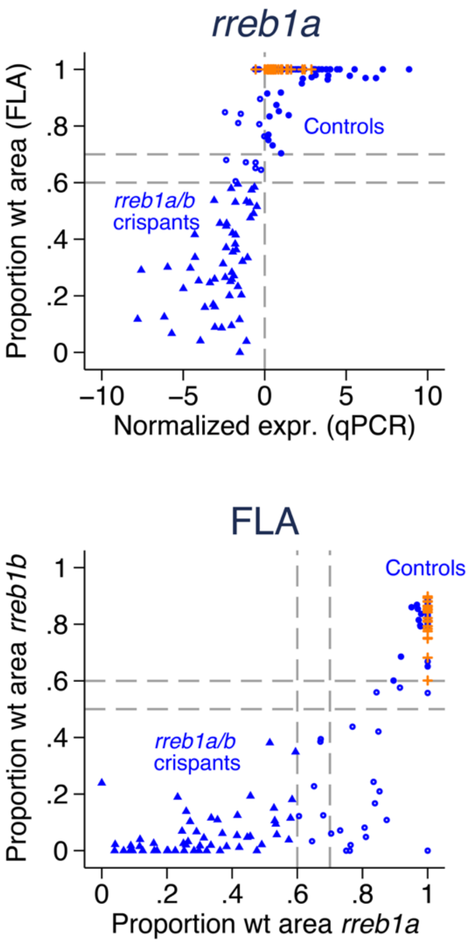

ESM Fig. 2: Results from fragment length analysis and qPCR for 126 microinjected zebrafish larvae (10 dpf) and 32 un-injected controls.
Comparing results from fragment length analysis and qPCR performed on the same samples shows that un-injected controls (orange crosses) have 100% of rreb1a (top left) and >60% of rreb1b (top right) peak area at the expected wildtype length. For both zebrafish genes, normalised gene expression based on qPCR results (i.e. residuals of expression at the CRISPR-targeted site adjusted for expression at a later exon) is >0 for most samples. Based on these results, we used fragment length analysis results for both genes (bottom left) to allocate larvae to the control group (blue triangles) if >70% of the rreb1a peak area and >60% of the rreb1b peak area was wildtype; while larvae with ≤60% and ≤50% of peak areas being wildtype for rreb1a and rreb1b were assigned to the rreb1a/b crispant group (filled blue circles). Larvae not fulfilling either criterion were excluded from the analysis (open blue circles).

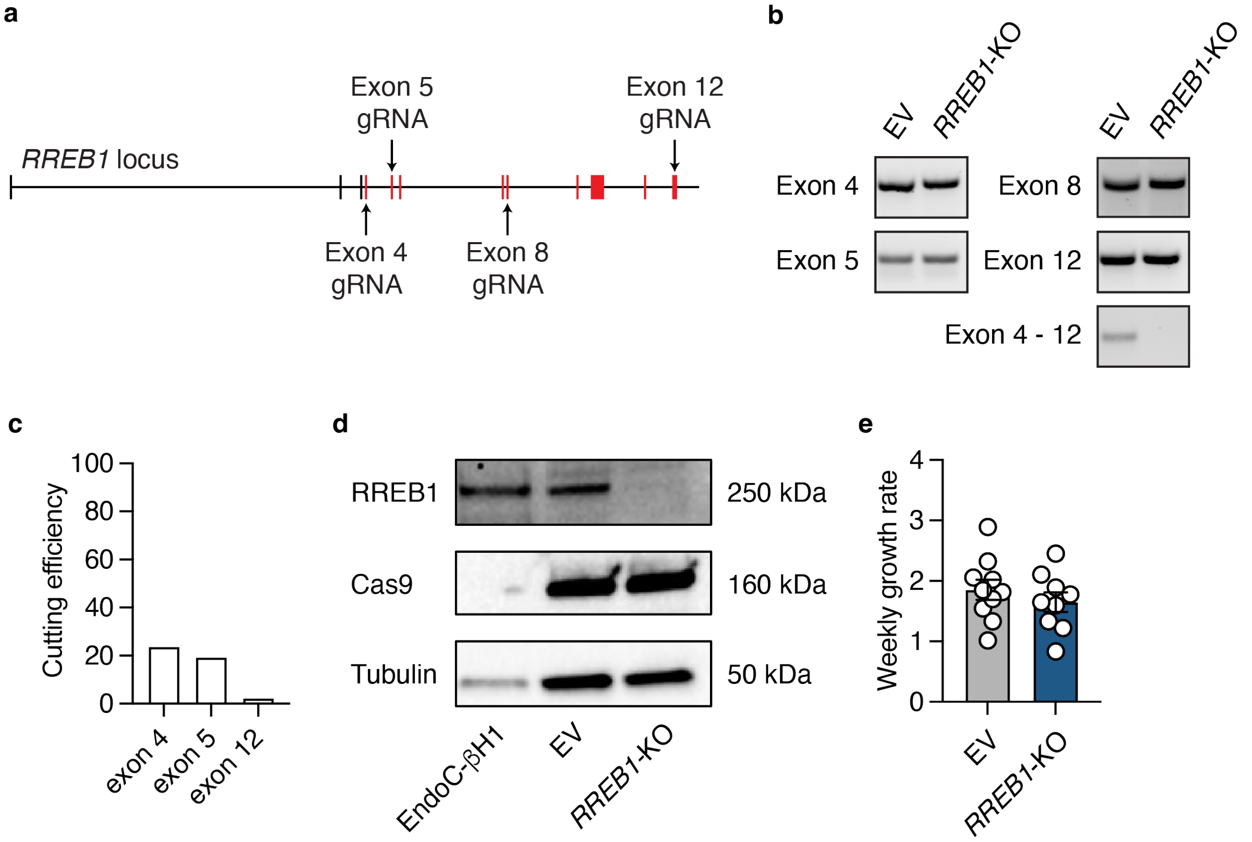

ESM Fig. 3: Generation and characterisation of *RREB1-* KO EndoC-βH1 cells.
(**a**) Schematic highlighting the genomic locations of the four sgRNAs used to generate *RREB1*-KO EndoC-βH1 cells. (**b**) Genomic PCR amplification of exon 4, 5, 8, 12, and exon 4 through 12 deletion in EV and *RREB1*-KO EndoC-βH1 cells. (**c**) Editing efficiency of individual sgRNAs in pooled *RREB1*-KO EndoC-βH1 cells. (**d**) Western blot for RREB1 (250 kDa), Cas9 (160 kDa), and Tubulin (50 kDa) in parental EndoC-βH1, EV control cells, and *RREB1*-KO knockout cells. (**e**) Weekly growth rate of EndoC-βH1 EV and *RREB1*-KO cells.

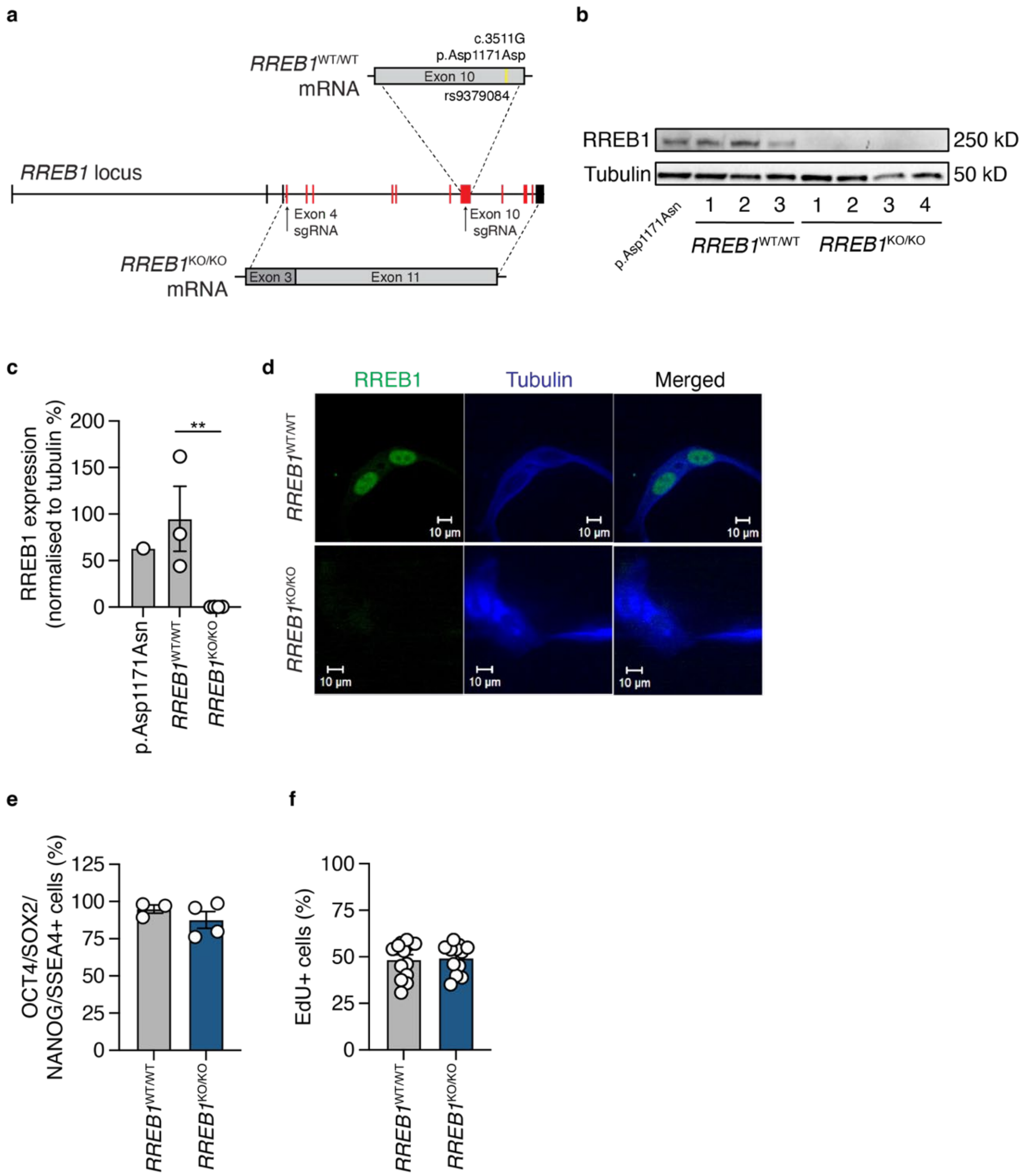

ESM Fig. 4: Generation and characterisation of *RREB1*$^{KO/KO}$ hiPSC lines.
(**a**) Schematic highlighting the genomic locations of the two sgRNAs used to generate *RREB1*$^{KO/KO}$ hiPSC lines by deleting ~50 kb of the *RREB1* gene. Located in exon 10 is rs9379084 (yellow) that was genetically engineered to be homozygous for the major p.Asp1171 allele. (**b-c**) Western blot and quantification for RREB1 (250 kDa) and Tubulin (50 kDa) in p.Asp1171Asn, *RREB1*$^{WT/WT}$ and *RREB1*$^{KO/KO}$ hiPSC lines. (**d**) Immunofluorescence staining of RREB1 (green) and tubulin (blue) in *RREB1*$^{WT/WT}$ and *RREB1*$^{KO/KO}$ hiPSC lines. (**e**) Quantification of percent of hiPSC cells expressing pluripotency proteins OCT4, SOX2, NANOG and SSEA4. (**f**) Quantification of the number of EdU+ hiPSCs. (Unpaired t-test)

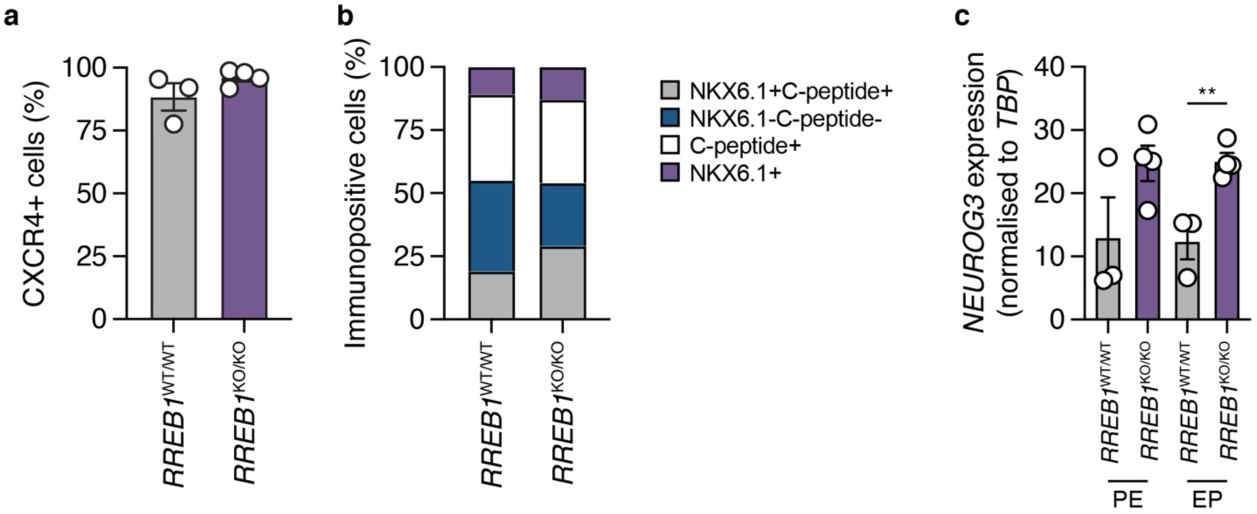

ESM Fig. 5: Characterisation of *in vitro* differentiation of *RREB1*$^{KO/KO}$ and *RREB1*$^{WT/WT}$ clones towards beta-like cells.

(**a**) Flow cytometric quantification of CXCR4+ definitive endoderm cells derived from *RREB1*$^{KO/KO}$ and *RREB1*$^{WT/WT}$ hiPSC lines. (**b**) Proportion BLCs of derived from *RREB1*$^{WT/WT}$ and *RREB1*$^{KO/KO}$ hiPSC cells immunopositive for NKX6.1+C-peptide+, NKX6.1-C-peptide-, C-peptide+, or NKX6.1+. (**c**) Expression of *NEUROG3* transcript in *RREB1*$^{KO/KO}$ and *RREB1*$^{WT/WT}$ hiPSC-derived pancreatic endoderm (PE) and endocrine progenitor (EP) cells. n=3-4. Data are presented as mean±SEM. p**<0.01. (Unpaired t-test)

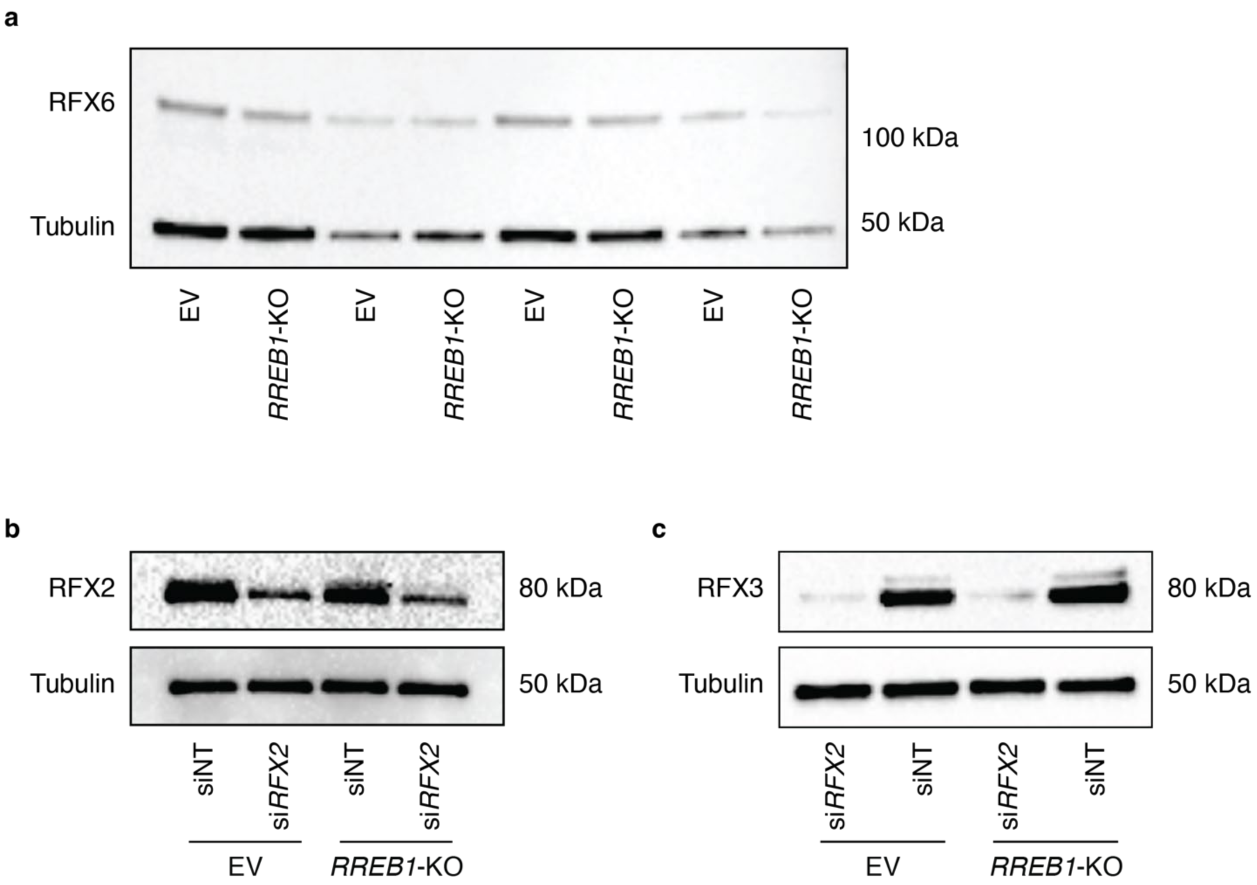

ESM Fig. 6: Expression of RFX proteins in knockdown and knockout RREB1 EndoC-βH1 cells.
(**a**) Protein expression of RFX6 in EV and *RREB*1-KO EndoC-βH1 cells. (**b-c**) Protein expression of (**b**) RFX2 and (**c**) RFX3 in siNT, si*RFX2*, and si*RFX3* treated EV and *RREB*1-KO EndoC-βH1 cells.

Human Islets checklist

Islet preparation	1	2	3	4	5	6	7	8[a]
**MANDATORY INFORMATION**								
Unique identifier	R029	R030	R032	R033	R034	R036	R037	R038
Donor age (years)	33	80	36	10	76	42	48	51
Donor sex (M/F)	Female	Female	Male	Female	Female	Male	Male	Male
Donor BMI (kg/m^2)	27.3	21.9	25.9	16.8	23.7	23.1	32.9	30.8
Donor HbA_{1c} or other measure of blood glucose control	no data	no data	no data	no data	6.2	no data	no data	no data
Origin/source of islets[b]	Alberta IsletCore	Alberta IsletCore	Alberta IsletCore	Alberta IsletCore	Alberta IsletCore	Alberta IsletCore	Alberta IsletCore	Alberta IsletCore
Islet isolation centre	Alberta IsletCore	Alberta IsletCore	Alberta IsletCore	Alberta IsletCore	Alberta IsletCore	Alberta IsletCore	Alberta IsletCore	Alberta IsletCore
Donor history of diabetes? Please select yes/no from drop down list	NO	NO	NO	NO	NO	NO	NO	NO

Islet preparation	9	10	11	12	13	14	15	16
**MANDATORY INFORMATION**								
Unique identifier	R039	R040	R041	R042	R044	R045	R047	R049
Donor age (years)	46	50	30	44	38	27	56	79
Donor sex (M/F)	Male	Female	Female	Male	Male	Female	Female	Male
Donor BMI (kg/m^2)	36.0	27.7	15.4	35.1	24.7	19.5	32.0	21.3
Donor HbA_{1c} or other measure of blood glucose control	5.9	5.9	5.7	no data	5.9	5.2	no data	no data

Origin/source of islets[b]	Alberta IsletCore	Alberta IsletCore	Alberta IsletCore	Alberta IsletCore	Alberta IsletCore	Alberta IsletCore	Alberta IsletCore	Alberta IsletCore
Islet isolation centre	Alberta IsletCore	Alberta IsletCore	Alberta IsletCore	Alberta IsletCore	Alberta IsletCore	Alberta IsletCore	Alberta IsletCore	Alberta IsletCore
Donor history of diabetes? Please select yes/no from drop down list	NO	NO	NO	NO	NO	NO	NO	NO

**Islet preparation**	**17**	**18**	**19**	**20**	**21**	**22**	**23**	**24**
**MANDATORY INFORMATION**								
Unique identifier	R050	R051	R052	R055	R056	R058	R060	R061
Donor age (years)	72	73	48	67	33	79	54	63
Donor sex (M/F)	Female	Male	Male	Female	Male	Female	Female	Male
Donor BMI (kg/m^2)	40.4	23.7	29.1	25.7	28.7	23.8	21.1	37.6
Donor HbA_{1c} or other measure of blood glucose control	5.8	5.7	no data	6	4.8	5.8	5.8	5.7
Origin/source of islets[b]	Alberta IsletCore	Alberta IsletCore	Alberta IsletCore	Alberta IsletCore	Alberta IsletCore	Alberta IsletCore	Alberta IsletCore	Alberta IsletCore
Islet isolation centre	Alberta IsletCore	Alberta IsletCore	Alberta IsletCore	Alberta IsletCore	Alberta IsletCore	Alberta IsletCore	Alberta IsletCore	Alberta IsletCore
Donor history of diabetes? Please select yes/no from drop down list	NO	NO	NO	NO	NO	NO	NO	NO

**Islet preparation**	**25**	**26**	**27**	**28**	**29**	**30**	**31**	**32**
**MANDATORY INFORMATION**								
Unique identifier	R065	R066	R067	R072	R073	R074	R075	R076

Donor age (years)	38	44	60	54	74	55	27	63
Donor sex (M/F)	Male	Male	Male	Female	Female	Male	Male	Female
Donor BMI (kg/m^2)	30.0	32.2	26.0	30.6	28.3	26.4	26.2	26.5
Donor HbA_{1c} or other measure of blood glucose control	no data	no data	5.5	6.2	5.4	5.5	5.4	no data
Origin/source of islets[b]	Alberta IsletCore	Alberta IsletCore	Alberta IsletCore	Alberta IsletCore	Alberta IsletCore	Alberta IsletCore	Alberta IsletCore	Alberta IsletCore
Islet isolation centre	Alberta IsletCore	Alberta IsletCore	Alberta IsletCore	Alberta IsletCore	Alberta IsletCore	Alberta IsletCore	Alberta IsletCore	Alberta IsletCore
Donor history of diabetes? Please select yes/no from drop down list	NO	NO	NO	NO	NO	NO	NO	NO

**Islet preparation**	**33**	**34**	**35**	**36**	**37**	**38**	**39**	**40**
**MANDATORY INFORMATION**								
Unique identifier	R077	R081	R082	R084	R085	R087	R088	R089
Donor age (years)	54	68	65	63	61	69	20	63
Donor sex (M/F)	Female	Male	Female	Female	Male	Male	Male	Female
Donor BMI (kg/m^2)	24.7	23.7	24.9	30.3	30.5	22.4	40.9	20.1
Donor HbA_{1c} or other measure of blood glucose control	5.2	5.9	5.4	5.5	5.6	5.9	5.8	no data
Origin/source of islets[b]	Alberta IsletCore	Alberta IsletCore	Alberta IsletCore	Alberta IsletCore	Alberta IsletCore	Alberta IsletCore	Alberta IsletCore	Alberta IsletCore
Islet isolation centre	Alberta IsletCore	Alberta IsletCore	Alberta IsletCore	Alberta IsletCore	Alberta IsletCore	Alberta IsletCore	Alberta IsletCore	Alberta IsletCore

Donor history of diabetes? Please select yes/no from drop down list	NO	NO	NO	NO	NO	NO	NO	NO

Islet preparation	41	42	43	44	45	46	47	48
**MANDATORY INFORMATION**								
Unique identifier	R091	R092	R094	R096	R098	R099	R100	R101
Donor age (years)	65	77	46	53	62	34	80	59
Donor sex (M/F)	Female	Male	Female	Female	Female	Female	Male	Female
Donor BMI (kg/m^2)	25.5	20.7	28.1	18.8	21.1	39.0	19.7	25.9
Donor HbA_{1c} or other measure of blood glucose control	5.6	5.7	5.8	5	6.3	5.5	5.2	5.5
Origin/source of islets[b]	Alberta IsletCore	Alberta IsletCore	Alberta IsletCore	Alberta IsletCore	Alberta IsletCore	Alberta IsletCore	Alberta IsletCore	Alberta IsletCore
Islet isolation centre	Alberta IsletCore	Alberta IsletCore	Alberta IsletCore	Alberta IsletCore	Alberta IsletCore	Alberta IsletCore	Alberta IsletCore	Alberta IsletCore
Donor history of diabetes? Please select yes/no from drop down list	NO	NO	NO	NO	NO	NO	NO	NO

Islet preparation	49	50	51	52	53	54	55	56
**MANDATORY INFORMATION**								
Unique identifier	R102	R104	R105	R106	R108	R109	R112	R113
Donor age (years)	54	42	59	52	59	60	54	62
Donor sex (M/F)	Male	Male	Female	Male	Female	Female	Female	Male
Donor BMI (kg/m^2)	41.4	27.1	35.1	19.9	24.8	25.0	32.9	30.5

Donor HbA_{1c} or other measure of blood glucose control	5.4	5.4	5.5	5.6	5.4	6.1	5.5	5.7
Origin/source of islets[b]	Alberta IsletCore	Alberta IsletCore	Alberta IsletCore	Alberta IsletCore	Alberta IsletCore	Alberta IsletCore	Alberta IsletCore	Alberta IsletCore
Islet isolation centre	Alberta IsletCore	Alberta IsletCore	Alberta IsletCore	Alberta IsletCore	Alberta IsletCore	Alberta IsletCore	Alberta IsletCore	Alberta IsletCore
Donor history of diabetes? Please select yes/no from drop down list	NO	NO	NO	NO	NO	NO	NO	NO

**Islet preparation**	**57**	**58**	**59**	**60**	**61**	**62**	**63**	**64**
**MANDATORY INFORMATION**								
Unique identifier	R114	R115	R116	R117	R120	R121	R122	R123
Donor age (years)	65	62	43	64	60	18	55	43
Donor sex (M/F)	Female	Female	Male	Male	Male	Male	Male	Female
Donor BMI (kg/m^2)	33.1	19.0	27.7	25.1	31.9	19.1	28.7	21.3
Donor HbA_{1c} or other measure of blood glucose control	6	5.5	no data	5.8	5.8	no data	5.9	no data
Origin/source of islets[b]	Alberta IsletCore	Alberta IsletCore	Alberta IsletCore	Alberta IsletCore	Alberta IsletCore	Alberta IsletCore	Alberta IsletCore	Alberta IsletCore
Islet isolation centre	Alberta IsletCore	Alberta IsletCore	Alberta IsletCore	Alberta IsletCore	Alberta IsletCore	Alberta IsletCore	Alberta IsletCore	Alberta IsletCore
Donor history of diabetes? Please select yes/no from drop down list	NO	NO	NO	NO	NO	NO	NO	NO

Islet preparation	65	66	67	68	69	70	71	72
**MANDATORY INFORMATION**								
Unique identifier	R124	R126	R128	R130	R134	R139	R140	R142
Donor age (years)	43	38	56	52	23	50	49	63
Donor sex (M/F)	Female	Male	Female	Male	Male	Male	Female	Female
Donor BMI (kg/m^2)	24.6	24.1	32.0	23.8	21.9	24.8	22.0	25.3
Donor HbA_{1c} or other measure of blood glucose control	5.2	5.2	6.1	5.3	5.9	5.9	5.6	4.9
Origin/source of islets[b]	Alberta IsletCore	Alberta IsletCore	Alberta IsletCore	Alberta IsletCore	Alberta IsletCore	Alberta IsletCore	Alberta IsletCore	Alberta IsletCore
Islet isolation centre	Alberta IsletCore	Alberta IsletCore	Alberta IsletCore	Alberta IsletCore	Alberta IsletCore	Alberta IsletCore	Alberta IsletCore	Alberta IsletCore
Donor history of diabetes? Please select yes/no from drop down list	NO	NO	NO	NO	NO	NO	NO	NO

Islet preparation	73	74	75	76	77	78	79	80
**MANDATORY INFORMATION**								
Unique identifier	R144	R145	R146	R147	R148	R149	R150	R151
Donor age (years)	50	55	52	62	40	47	42	46
Donor sex (M/F)	Female	Female	Male	Male	Male	Male	Male	Female
Donor BMI (kg/m^2)	35.4	24.1	25.3	26.0	29.1	41.2	31.7	26.7
Donor HbA_{1c} or other measure of blood glucose control	5	5.5	5.5	5.4	no data	5.9	5.9	5.4
Origin/source of islets[b]	Alberta IsletCore	Alberta IsletCore	Alberta IsletCore	Alberta IsletCore	Alberta IsletCore	Alberta IsletCore	Alberta IsletCore	Alberta IsletCore

Islet isolation centre	Alberta IsletCore	Alberta IsletCore	Alberta IsletCore	Alberta IsletCore	Alberta IsletCore	Alberta IsletCore	Alberta IsletCore	Alberta IsletCore
Donor history of diabetes? Please select yes/no from drop down list	NO	NO	NO	NO	NO	NO	NO	NO

**Islet preparation**	**81**	**82**	**83**	**84**	**85**	**86**	**87**	**88**
**MANDATORY INFORMATION**								
Unique identifier	R153	R156	R157	R159	R161	R162	R163	R164
Donor age (years)	53	69	60	60	71	74	15	44
Donor sex (M/F)	Female	Female	Female	Male	Female	Female	Female	Female
Donor BMI (kg/m^2)	21.1	28.8	23.3	30.4	19.7	28.6	23.4	30.8
Donor HbA_{1c} or other measure of blood glucose control	5.4	6.2	6	5.5	6.3	6.2	5.1	6
Origin/source of islets[b]	Alberta IsletCore	Alberta IsletCore	Alberta IsletCore	Alberta IsletCore	Alberta IsletCore	Alberta IsletCore	Alberta IsletCore	Alberta IsletCore
Islet isolation centre	Alberta IsletCore	Alberta IsletCore	Alberta IsletCore	Alberta IsletCore	Alberta IsletCore	Alberta IsletCore	Alberta IsletCore	Alberta IsletCore
Donor history of diabetes? Please select yes/no from drop down list	NO	NO	NO	NO	NO	NO	NO	NO

Islet preparation	89	90	91	92	93	94	95	96
**MANDATORY INFORMATION**								
Unique identifier	R165	R167	R168	R169	R174	R175	R176	R177
Donor age (years)	30	84	44	60	44	53	55	28
Donor sex (M/F)	Male	Female	Male	Male	Male	Female	Male	Male
Donor BMI (kg/m^2)	23.9	21.1	24.1	23.8	34.4	29.1	23.6	23.4
Donor HbA_{1c} or other measure of blood glucose control	5.9	no data	5.4	5.7	4.7	5.4	5.8	no data
Origin/source of islets[b]	Alberta IsletCore	Alberta IsletCore	Alberta IsletCore	Alberta IsletCore	Alberta IsletCore	Alberta IsletCore	Alberta IsletCore	Alberta IsletCore
Islet isolation centre	Alberta IsletCore	Alberta IsletCore	Alberta IsletCore	Alberta IsletCore	Alberta IsletCore	Alberta IsletCore	Alberta IsletCore	Alberta IsletCore
Donor history of diabetes? Please select yes/no from drop down list	NO	NO	NO	NO	NO	NO	NO	NO

Islet preparation	97	98	99	100	101	102	103	104
**MANDATORY INFORMATION**								
Unique identifier	R178	R179	R180	R181	R184	R189	R190	R193
Donor age (years)	54	61	68	42	59	50	66	69
Donor sex (M/F)	Female	Male	Female	Female	Female	Male	Female	Male
Donor BMI (kg/m^2)	25.3	25.1	21.7	29.4	21.5	28.2	33.1	39.5
Donor HbA_{1c} or other measure of blood glucose control	no data	5.7	5.9	6	5.2	5.4	6.2	6.2
Origin/source of islets[b]	Alberta IsletCore	Alberta IsletCore	Alberta IsletCore	Alberta IsletCore	Alberta IsletCore	Alberta IsletCore	Alberta IsletCore	Alberta IsletCore

Islet isolation centre	Alberta IsletCore	Alberta IsletCore	Alberta IsletCore	Alberta IsletCore	Alberta IsletCore	Alberta IsletCore	Alberta IsletCore	Alberta IsletCore
Donor history of diabetes? Please select yes/no from drop down list	NO	NO	NO	NO	NO	NO	NO	NO

Islet preparation	105	106	107	108	109	110	111	112
**MANDATORY INFORMATION**								
Unique identifier	R194	R200	R202	R203	R204	R205	R207	R208
Donor age (years)	54	65	68	50	62	53	50	27
Donor sex (M/F)	Male	Male	Female	Male	Male	Male	Female	Male
Donor BMI (kg/m^2)	32.3	27.1	25.2	44.4	23.8	29.6	22.2	29.0
Donor HbA_{1c} or other measure of blood glucose control	5.7	5.1	5.7	5.7	5.6	5.9	no data	5.5
Origin/source of islets[b]	Alberta IsletCore	Alberta IsletCore	Alberta IsletCore	Alberta IsletCore	Alberta IsletCore	Alberta IsletCore	Alberta IsletCore	Alberta IsletCore
Islet isolation centre	Alberta IsletCore	Alberta IsletCore	Alberta IsletCore	Alberta IsletCore	Alberta IsletCore	Alberta IsletCore	Alberta IsletCore	Alberta IsletCore
Donor history of diabetes? Please select yes/no from drop down list	NO	NO	NO	NO	NO	NO	NO	NO

Islet preparation	113	114	115	116	117	118	119	120
**MANDATORY INFORMATION**								
Unique identifier	R210	R215	R216	R217	R218	R219	R221	R223
Donor age (years)	46	51	38	71	73	53	44	54
Donor sex (M/F)	Male	Male	Male	Female	Female	Male	Male	Male

Donor BMI (kg/m^2)	24.5	29.4	42.6	35.5	28.4	26.0	30.5	27.0
Donor HbA_{1c} or other measure of blood glucose control	5.3	6	5.6	6.3	5.9	no data	5.3	5.8
Origin/source of islets[b]	Alberta IsletCore	Alberta IsletCore	Alberta IsletCore	Alberta IsletCore	Alberta IsletCore	Alberta IsletCore	Alberta IsletCore	Alberta IsletCore
Islet isolation centre	Alberta IsletCore	Alberta IsletCore	Alberta IsletCore	Alberta IsletCore	Alberta IsletCore	Alberta IsletCore	Alberta IsletCore	Alberta IsletCore
Donor history of diabetes? Please select yes/no from drop down list	NO	NO	NO	NO	NO	NO	NO	NO

**Islet preparation**	**121**	**122**	**123**	**124**	**125**	**126**	**127**	**128**
**MANDATORY INFORMATION**								
Unique identifier	R224	R226	R227	R228	R230	R232	R233	R234
Donor age (years)	30	30	32	45	58	66	76	50
Donor sex (M/F)	Male	Female	Male	Female	Male	Female	Female	Female
Donor BMI (kg/m^2)	33.3	32.3	19.9	29.6	29.4	18.5	19.3	31.7
Donor HbA_{1c} or other measure of blood glucose control	no data	4.9	4.9	4.5	6.2	6.1	6	5.7
Origin/source of islets[b]	Alberta IsletCore	Alberta IsletCore	Alberta IsletCore	Alberta IsletCore	Alberta IsletCore	Alberta IsletCore	Alberta IsletCore	Alberta IsletCore
Islet isolation centre	Alberta IsletCore	Alberta IsletCore	Alberta IsletCore	Alberta IsletCore	Alberta IsletCore	Alberta IsletCore	Alberta IsletCore	Alberta IsletCore
Donor history of diabetes? Please select yes/no from	NO	NO	NO	NO	NO	NO	NO	NO

drop down list								

Islet preparation	129	130	131	132	133	134	135	136
**MANDATORY INFORMATION**								
Unique identifier	R235	R237	R238	R243	R245	R246	R247	R248
Donor age (years)	53	61	52	39	63	65	72	29
Donor sex (M/F)	Female	Male	Male	Male	Male	Female	Male	Male
Donor BMI (kg/m^2)	24.5	19.7	26.1	27.1	22.3	39.2	23.9	23.7
Donor HbA_{1c} or other measure of blood glucose control	5.7	5.9	5.7	5.8	5.6	5.8	no data	6.1
Origin/source of islets[b]	Alberta IsletCore	Alberta IsletCore	Alberta IsletCore	Alberta IsletCore	Alberta IsletCore	Alberta IsletCore	Alberta IsletCore	Alberta IsletCore
Islet isolation centre	Alberta IsletCore	Alberta IsletCore	Alberta IsletCore	Alberta IsletCore	Alberta IsletCore	Alberta IsletCore	Alberta IsletCore	Alberta IsletCore
Donor history of diabetes? Please select yes/no from drop down list	NO	NO	NO	NO	NO	NO	NO	NO

Islet preparation	137	138	139	140	141	142	143	144
**MANDATORY INFORMATION**								
Unique identifier	R249	R250	R253	R254	R256	R266	R267	R269
Donor age (years)	62	58	57	62	23	74	67	14
Donor sex (M/F)	Female	Male	Male	Male	Male	Female	Female	Male
Donor BMI (kg/m^2)	22.2	33.0	25.6	20.2	32.5	29.2	23.7	21.5

Donor HbA_{1c} or other measure of blood glucose control	no data	6.2	5	6.3	5.4	6	6.3	no data
Origin/source of islets[b]	Alberta IsletCore	Alberta IsletCore	Alberta IsletCore	Alberta IsletCore	Alberta IsletCore	Alberta IsletCore	Alberta IsletCore	Alberta IsletCore
Islet isolation centre	Alberta IsletCore	Alberta IsletCore	Alberta IsletCore	Alberta IsletCore	Alberta IsletCore	Alberta IsletCore	Alberta IsletCore	Alberta IsletCore
Donor history of diabetes? Please select yes/no from drop down list	NO	NO	NO	NO	NO	NO	NO	NO

**Islet preparation**	**145**	**146**	**147**	**148**	**149**	**150**	**151**	**152**
**MANDATORY INFORMATION**								
Unique identifier	R270	R271	R272	R274	R275	R277	R278	R279
Donor age (years)	35	60	56	42	46	47	57	31
Donor sex (M/F)	Female	Female	Male	Male	Male	Male	Male	Male
Donor BMI (kg/m^2)	25.5	26.0	26.9	25.2	28.7	33.8	27.6	22.6
Donor HbA_{1c} or other measure of blood glucose control	5.3	5.5	5.6	no data	4.5	5.6	5.7	5
Origin/source of islets[b]	Alberta IsletCore	Alberta IsletCore	Alberta IsletCore	Alberta IsletCore	Alberta IsletCore	Alberta IsletCore	Alberta IsletCore	Alberta IsletCore
Islet isolation centre	Alberta IsletCore	Alberta IsletCore	Alberta IsletCore	Alberta IsletCore	Alberta IsletCore	Alberta IsletCore	Alberta IsletCore	Alberta IsletCore
Donor history of diabetes? Please select yes/no from drop down list	NO	NO	NO	NO	NO	NO	NO	NO

Islet preparation	153	154	155	156	157	158	159	160
**MANDATORY INFORMATION**								
Unique identifier	R281	R282	R283	R284	R285	R286	R288	R290
Donor age (years)	30	57	22	32	19	41	69	74
Donor sex (M/F)	Female	Male	Male	Female	Male	Male	Female	Female
Donor BMI (kg/m^2)	25.1	26.4	22.5	30.1	21.6	20.4	27.7	35.8
Donor HbA_{1c} or other measure of blood glucose control	no data	6	no data	5.9	5.4	5.2	5.7	5.9
Origin/source of islets[b]	Alberta IsletCore	Alberta IsletCore	Alberta IsletCore	Alberta IsletCore	Alberta IsletCore	Alberta IsletCore	Alberta IsletCore	Alberta IsletCore
Islet isolation centre	Alberta IsletCore	Alberta IsletCore	Alberta IsletCore	Alberta IsletCore	Alberta IsletCore	Alberta IsletCore	Alberta IsletCore	Alberta IsletCore
Donor history of diabetes? Please select yes/no from drop down list	NO	NO	NO	NO	NO	NO	NO	NO

Islet preparation	161	162	163	164	165	166	167	168
**MANDATORY INFORMATION**								
Unique identifier	R291	R292	R294	R297	R299	R300	R301	R302
Donor age (years)	40	47	32	69	44	30	18	62
Donor sex (M/F)	Female	Male	Male	Male	Female	Female	Male	Female
Donor BMI (kg/m^2)	25.5	27.6	27.8	27.2	25.4	25.3	19	32.3
Donor HbA_{1c} or other measure of blood glucose control	6	5.6	5.7	no data	4.7	no data	5	6.2
Origin/source of islets[b]	Alberta IsletCore	Alberta IsletCore	Alberta IsletCore	Alberta IsletCore	Alberta IsletCore	Alberta IsletCore	Alberta IsletCore	Alberta IsletCore

Islet isolation centre	Alberta IsletCore	Alberta IsletCore	Alberta IsletCore	Alberta IsletCore	Alberta IsletCore	Alberta IsletCore	Alberta IsletCore	Alberta IsletCore
Donor history of diabetes? Please select yes/no from drop down list	NO	NO	NO	NO	NO	NO	NO	NO

**Islet preparation**	**169**	**170**	**171**	**172**	**173**	**174**	**175**	**176**
**MANDATORY INFORMATION**								
Unique identifier	R303	R304	R305	R306	R308	R309	R310	R313
Donor age (years)	56	46	60	22	20	47	25	41
Donor sex (M/F)	Female	Male	Male	Female	Male	Female	Male	Male
Donor BMI (kg/m^2)	24.1	21.5	21	21.1	19.8	27.4	26.4	29
Donor HbA_{1c} or other measure of blood glucose control	no data	no data	5.6	5.3	5.5	5.5	5.4	5.5
Origin/source of islets[b]	Alberta IsletCore	Alberta IsletCore	Alberta IsletCore	Alberta IsletCore	Alberta IsletCore	Alberta IsletCore	Alberta IsletCore	Alberta IsletCore
Islet isolation centre	Alberta IsletCore	Alberta IsletCore	Alberta IsletCore	Alberta IsletCore	Alberta IsletCore	Alberta IsletCore	Alberta IsletCore	Alberta IsletCore
Donor history of diabetes? Please select yes/no from drop down list	NO	NO	NO	NO	NO	NO	NO	NO

**Islet preparation**	**177**	**178**	**179**	**180**	**181**	**182**	**183**	**184**
**MANDATORY INFORMATION**								
Unique identifier	R314	R316	R317	R318	R319	R320	R321	R322
Donor age (years)	31	52	54	54	68	58	25	44
Donor sex (M/F)	Female	Male	Male	Male	Male	Male	Female	Female

Donor BMI (kg/m^2)	30.3	26	26.4	20.5	27.8	19.9	31.6	23.2
Donor HbA$_{1c}$ or other measure of blood glucose control	5	5.7	5.1	5	5	no data	4.5	4.9
Origin/source of islets[b]	Alberta IsletCore	Alberta IsletCore	Alberta IsletCore	Alberta IsletCore	Alberta IsletCore	Alberta IsletCore	Alberta IsletCore	Alberta IsletCore
Islet isolation centre	Alberta IsletCore	Alberta IsletCore	Alberta IsletCore	Alberta IsletCore	Alberta IsletCore	Alberta IsletCore	Alberta IsletCore	Alberta IsletCore
Donor history of diabetes? Please select yes/no from drop down list	NO	NO	NO	NO	NO	NO	NO	NO

**Islet preparation**	**185**	**186**	**187**	**188**	**189**	**190**	**191**	**192**
**MANDATORY INFORMATION**								
Unique identifier	R323	R324	R325	R326	R327	R328	R330	R332
Donor age (years)	60	58	50	26	57	48	39	64
Donor sex (M/F)	Female	Male	Male	Male	Male	Male	Female	Male
Donor BMI (kg/m^2)	24.9	36.6	30.3	27	33.9	22.7	24.2	29.6
Donor HbA$_{1c}$ or other measure of blood glucose control	5.1	5.1	no data	5.5	5.8	5.2	5.1	4.8
Origin/source of islets[b]	Alberta IsletCore	Alberta IsletCore	Alberta IsletCore	Alberta IsletCore	Alberta IsletCore	Alberta IsletCore	Alberta IsletCore	Alberta IsletCore
Islet isolation centre	Alberta IsletCore	Alberta IsletCore	Alberta IsletCore	Alberta IsletCore	Alberta IsletCore	Alberta IsletCore	Alberta IsletCore	Alberta IsletCore
Donor history of diabetes? Please select yes/no from	NO	NO	NO	NO	NO	NO	NO	NO

drop down list								

Islet preparation	193	194	195	196	197	198	199	200
MANDATORY INFORMATION								
Unique identifier	R333	R334	R335	R338	R340	R341	R342	R343
Donor age (years)	23	50	25	30	36	42	35	53
Donor sex (M/F)	Male	Female	Male	Male	Male	Male	Male	Female
Donor BMI (kg/m^2)	24	31.2	24.6	25.5	23.3	30	25.1	32.8
Donor HbA_{1c} or other measure of blood glucose control	5	5.8	4.9	5.3	5.3	no data	5.5	5.7
Origin/source of islets[b]	Alberta IsletCore	Alberta IsletCore	Alberta IsletCore	Alberta IsletCore	Alberta IsletCore	Alberta IsletCore	Alberta IsletCore	Alberta IsletCore
Islet isolation centre	Alberta IsletCore	Alberta IsletCore	Alberta IsletCore	Alberta IsletCore	Alberta IsletCore	Alberta IsletCore	Alberta IsletCore	Alberta IsletCore
Donor history of diabetes? Please select yes/no from drop down list	NO	NO	NO	NO	NO	NO	NO	NO

Islet preparation	201	202	203	204	205	206	207	208
MANDATORY INFORMATION								
Unique identifier	R344	R346	R348	R350	R351	R352	R353	R354
Donor age (years)	59	27	43	38	51	64	69	75
Donor sex (M/F)	Male	Male	Female	Female	Male	Female	Male	Male
Donor BMI (kg/m^2)	27.8	24.6	16.4	21.7	21.4	20.4	22.7	30.2

Donor HbA_{1c} or other measure of blood glucose control	5.5	5.7	5.7	5.5	4.8	5.3	no data	5.2
Origin/source of islets[b]	Alberta IsletCore	Alberta IsletCore	Alberta IsletCore	Alberta IsletCore	Alberta IsletCore	Alberta IsletCore	Alberta IsletCore	Alberta IsletCore
Islet isolation centre	Alberta IsletCore	Alberta IsletCore	Alberta IsletCore	Alberta IsletCore	Alberta IsletCore	Alberta IsletCore	Alberta IsletCore	Alberta IsletCore
Donor history of diabetes? Please select yes/no from drop down list	NO	NO	NO	NO	NO	NO	NO	NO

**Islet preparation**	**209**	**210**	**211**	**212**	**213**	**214**	**215**	**216**
**MANDATORY INFORMATION**								
Unique identifier	R355	R356	R357	R360	R361	R362	R363	R364
Donor age (years)	79	45	64	51	65	54	31	58
Donor sex (M/F)	Male	Female	Male	Female	Female	Male	Male	Male
Donor BMI (kg/m^2)	23.5	29.7	24.3	26.1	20.8	29.4	31.9	27.8
Donor HbA_{1c} or other measure of blood glucose control	5.2	5.1	no data	4.9	5.3	no data	5.1	5.6
Origin/source of islets[b]	Alberta IsletCore	Alberta IsletCore	Alberta IsletCore	Alberta IsletCore	Alberta IsletCore	Alberta IsletCore	Alberta IsletCore	Alberta IsletCore
Islet isolation centre	Alberta IsletCore	Alberta IsletCore	Alberta IsletCore	Alberta IsletCore	Alberta IsletCore	Alberta IsletCore	Alberta IsletCore	Alberta IsletCore
Donor history of diabetes? Please select yes/no from drop down list	NO	NO	NO	NO	NO	NO	NO	NO

Islet preparation	217	218	219	220	221	222	223	224
MANDATORY INFORMATION								
Unique identifier	R365	R366	R367	R368	R369	R370	R371	R372
Donor age (years)	31	31	58	60	66	12	55	58
Donor sex (M/F)	Female	Male	Female	Male	Male	Male	Female	Male
Donor BMI (kg/m^2)	20.3	26.2	30.5	17.7	25.6	20.8	29.1	33.2
Donor HbA$_{1c}$ or other measure of blood glucose control	4.8	5.3	5.6	5.1	4.9	5	5.2	6.1
Origin/source of islets[b]	Alberta IsletCore	Alberta IsletCore	Alberta IsletCore	Alberta IsletCore	Alberta IsletCore	Alberta IsletCore	Alberta IsletCore	Alberta IsletCore
Islet isolation centre	Alberta IsletCore	Alberta IsletCore	Alberta IsletCore	Alberta IsletCore	Alberta IsletCore	Alberta IsletCore	Alberta IsletCore	Alberta IsletCore
Donor history of diabetes? Please select yes/no from drop down list	NO	NO	NO	NO	NO	NO	NO	NO

Islet preparation	225	226	227	228	229	230	231	232
MANDATORY INFORMATION								
Unique identifier	R373	R374	R375	R377	R379	R382	R383	R385
Donor age (years)	31	34	28	56	41	45	49	47
Donor sex (M/F)	Male	Male	Male	Male	Male	Male	Male	Male
Donor BMI (kg/m^2)	27.5	24.5	24.9	24.3	34	29.7	25.8	31
Donor HbA$_{1c}$ or other measure of blood glucose control	4.4	5.1	4.5	5.3		5.5	4	5.1
Origin/source of islets[b]	Alberta IsletCore	Alberta IsletCore	Alberta IsletCore	Alberta IsletCore	Alberta IsletCore	Alberta IsletCore	Alberta IsletCore	Alberta IsletCore

Islet isolation centre	Alberta IsletCore	Alberta IsletCore	Alberta IsletCore	Alberta IsletCore	Alberta IsletCore	Alberta IsletCore	Alberta IsletCore	Alberta IsletCore
Donor history of diabetes? Please select yes/no from drop down list	NO	NO	NO	NO	NO	NO	NO	NO

Islet preparation	233	234	235	236	237	238	239
**MANDATORY INFORMATION**							
Unique identifier	R387	R388	R389	R390	R391	R392	R394
Donor age (years)	53	45	65	3	67	8	2
Donor sex (M/F)	Female	Male	Female	Male	Male	Female	Female
Donor BMI (kg/m^2)	31	33.8	24.4	19.8	24.5	15.9	18.9
Donor HbA_{1c} or other measure of blood glucose control	5.6	5.3	5.5	3.1	4.9	4.8	5.1
Origin/source of islets[b]	Alberta IsletCore	Alberta IsletCore	Alberta IsletCore	Alberta IsletCore	Alberta IsletCore	Alberta IsletCore	Alberta IsletCore
Islet isolation centre	Alberta IsletCore	Alberta IsletCore	Alberta IsletCore	Alberta IsletCore	Alberta IsletCore	Alberta IsletCore	Alberta IsletCore
Donor history of diabetes? Please select yes/no from drop down list	NO	NO	NO	NO	NO	NO	NO

References

[1] R Core Team (2022) R: A Language and Environment for Statistical Computing. Available from https://www.R-project.org
